# The Small Subunit 1 of the *Arabidopsis* Isopropylmalate Isomerase Is Required for Normal Growth and Development and the Early Stages of Glucosinolate Formation

**DOI:** 10.1371/journal.pone.0091071

**Published:** 2014-03-07

**Authors:** Janet Imhof, Florian Huber, Michael Reichelt, Jonathan Gershenzon, Christoph Wiegreffe, Kurt Lächler, Stefan Binder

**Affiliations:** 1 Institut Molekulare Botanik, Universität Ulm, Ulm, Germany; 2 Max Planck Institut für Chemische Ökologie, Abt. Biochemie, Beutenberg Campus, Jena, Germany; 3 Institut für Molekulare und Zelluläre Anatomie, Universität Ulm, Ulm, Germany; Universidade Federal de Vicosa, Brazil

## Abstract

In *Arabidopsis thaliana* the evolutionary and functional relationship between Leu biosynthesis and the Met chain elongation pathway, the first part of glucosinolate formation, is well documented. Nevertheless the exact functions of some pathway components are still unclear. Isopropylmalate isomerase (IPMI), an enzyme usually involved in Leu biosynthesis, is a heterodimer consisting of a large and a small subunit. While the large protein is encoded by a single gene (*ISOPROPYLMALATE ISOMERASE LARGE SUBUNIT1*), three genes encode small subunits (*ISOPROPYLMALATE ISOMERASE SMALL SUBUNIT1 to 3*). We have now analyzed small subunit 1 (ISOPROPYLMALATE ISOMERASE SMALL SUBUNIT1) employing artificial microRNA for a targeted knockdown of the encoding gene. Strong reduction of corresponding mRNA levels to less than 5% of wild-type levels resulted in a severe phenotype with stunted growth, narrow pale leaf blades with green vasculature and abnormal adaxial-abaxial patterning as well as anomalous flower morphology. Supplementation of the knockdown plants with leucine could only partially compensate for the morphological and developmental abnormalities. Detailed metabolite profiling of the knockdown plants revealed changes in the steady state levels of isopropylmalate and glucosinolates as well as their intermediates demonstrating a function of IPMI SSU1 in both leucine biosynthesis and the first cycle of Met chain elongation. Surprisingly the levels of free leucine slightly increased suggesting an imbalanced distribution of leucine within cells and/or within plant tissues.

## Introduction

Humans and other animals are not able to synthesize Leu, one of the essential amino acids, and thus depend on external sources for this aliphatic amino acid. Leu, Ile and Val form the small group of branched-chain amino acids (BCAA) that is characterized by short aliphatic side chains. Therefore these amino acids are often found in membrane-spanning domains of proteins [1].

In prokaryotes, fungi and plants, *de novo* BCAA synthesis follows a common scheme. Ile and Val are synthesized by a single set of enzymes that catalyze reactions with different substrates. While Ile is synthesized by a straight pathway, the biosynthetic route to Val branches at the level of the keto acid 3-methyl-2-oxobutanoate (3MOB). This intermediate can either be directly transaminated to Val or it can be used as substrate for the biosynthesis of Leu. The latter sequence includes three additional reactions catalyzed by isopropylmalate synthase (IPMS), isopropylmalate isomerase (IPMI) and isopropylmalate dehydrogenase (IPMDH) to form the elongated keto acid 4-methyl-2-oxopentanoate (4MOP), which is then converted into Leu [1]. In *Arabidopsis thaliana* (*Arabidopsis*) and other Brassicaceae, a similar pathway is also used for the chain elongation of Met. This cycle provides Met derivatives containing one or several additional methylene groups in their side chain for the biosynthesis of aliphatic glucosinolates [2]. The close relationship between these pathways is evident not only from the cascades of identical reaction types, but also from the fact that proteins active in these different pathways are encoded by the same gene families. In addition, there are enzymes that function in both pathways. One such enzyme is IPMI, a heterodimer made up of a large and a small subunit. In *Arabidopsis*, the large subunit (IPMI LSU1, also designated AtLeuC) is encoded by a single gene. IPMI LSU1 is involved in both Leu biosynthesis and Met chain elongation, while a functional specialization has been suggested for the three different small subunits (IPMI SSU1, 2 and 3 or AtLeuD3, 1 and 2). IPMI SSU2 and IPMI SSU3 take part in the Met chain elongation pathway, but up to now, no direct evidence has been obtained for the function of IPMI SSU1. The knockout of this gene interferes with female gametophyte development, which contributes to mutant lethality and prevents establishment of homozygous knockout mutants. This observation suggested that IPMI SSU1 has a crucial function most likely in the formation of Leu [3]–[5].

To learn more about the exact function of IPMI SSU1 and about its importance for growth and development in *Arabidopsis*, we used artificial microRNA (amiRNA) to specifically knockdown IPMI SSU1 expression (amiR-SSU1). Strong reduction of IPMI SSU1 transcript levels lead to a severe phenotype with stunted growth, narrow pale leaf blades, anomalous flower morphology and abnormal adaxial-abaxial patterning in leaves. While no changes were seen in the abundance of several plastid proteins, chloroplasts were smaller and contained less starch. Changes in the steady state levels of isopropylmalate and glucosinolates as well as intermediates of these secondary metabolites demonstrated a function of IPMI SSU1 in both Leu and glucosinolate metabolism.

## Materials and Methods

### Plant Material

*Arabidopsis thaliana* (*Arabidopsis*) plants were cultivated on standard soil supplemented with Vermiculite and Osmocote Exact Mini (Scotts) in a growth chamber under condition described previously [6]. An *ipmi ssu2-1*/*ipmi ssu3-1* double knockout was obtained by crossing the previously analyzed *ipmi ssu2-1* (Col-0) with *ipmi ssu3-1* (Ws) [5]. Crossing was performed following a standard procedure [7]. Administration of 2 mM Leu or 2 mM BCAA was started when sowing the seeds on soil. The amino acids were supplemented in a watering interval of three or four days. Seeds were also grown on plates containing 0.2 mM Leu or 0.2 mM BCAA in Murashige and Skoog (MS) medium without sucrose. Plant transformation was done via *Agrobacterium* strain GV2260 by floral dip [8], [9].

### Nucleic Acids Analysis

Total RNA from leaves was isolated using the Spectrum Plant Total RNA Kit (Sigma-Aldrich) whereas total DNA was extracted following a previously established protocol but with two isopropanol precipitations [10]. AmiRNA were designed using the Web MicroRNA Designer version 3.1 (WMD3) (http://wmd.weigelworld.org/cgi-bin/mirnatools.pl). Cloning of amiRNA constructs followed a strategy applying overlap PCRs with vector pRS300 containing the miRNA319a backbone and the primers listed in Table S1 [11]. The PCR products representing the amiRNA genes were then cloned into pMDC123 vectors containing a CaMV 35S promoter and NOS terminator using the *Sma*I/*Eco*RV and *Sac*I restriction sites. These constructs were introduced into *Arabidopsis* Col-0 plants as described in the previous section. Transgenic plants were selected by their resistance to Basta and the transcript levels of the different IPMI subunits were determined by semi-quantitative RT-PCR according to standard procedures [12]. To this end cDNA was synthesized from 2 µg of total RNA using M-MLV RT (H-) reverse transcriptase (Promega) and oligo(dT) primer DTXSC. PCR were then performed with oligonucleotide pairs SSU1comp.H/SSU1comp.R (*IPMI SSU1*), SSU2comp.H/SSU2comp.R (*IPMI SSU2*), SSU3comp.H/SSU3comp.R (*IPMI SSU3*), At4g13430-Bur.H/At4g13430-Bur.R (*IPMI LSU1*) and UBC9-H.2/At4g27960-2 (*UBIQUITIN9*). Real-Time quantitative RT-PCR was done with a DyNAmo Color Flash SYBR Green qPCR Kit as described before [13]. The level of the *IPMI SSU1* transcripts was determined with oligonucleotide pair At2g43090.H/At2g43090.R from three independent biological replicates. Oligonucleotide sequences are given in Table S1.

### Metabolite Profiling

Metabolite levels were measured in approximately three week-old rosette leaves or in seeds. Measurements in whole leaves were done in eight replicates (each replicate a pool of 6 to 10 plants) from a single cultivation per line or in eight seed pools (10 or 20 mg). For metabolite analysis in different parts of the leaves, midribs or leaf blades without midribs were obtained from 4 to 6 leaves per plant. Plant material harvested from ten different plants was pooled and free metabolites were determined in 5 or 6 pools. Amino acids were extracted and quantified by reversed phase-HPLC and fluorescence detection or by LC-MS/MS as described previously with minor modifications [14], [15]. Details on the amino acid analysis by LC-MS/MS can be found in Protocol S1.

In addition, the relative contents of isopropylmalate (IPM), 2-(2′-methylsulfinyl)ethylmalate (2MSEM), and 2-(3′-methylsulfinyl)propylmalate (2MSPM) were determined by an LC-MS/MS analysis. These compounds are IPMI products or oxidized derivatives; 2MSEM and 2MSPM are derived from the true enzyme products, 2-(2′-methylthio)ethylmalate and 2-(3′-methylthio)propyl)malate, respectively. A detailed description of this procedure is given in Protocol S2.

### Analysis of Phytohormones

Phytohormones were analyzed from the 80% methanolic extracts obtained during glucosinolate analysis spiked with internal standards by LC-MS/MS as described before with the modification that an API5000 mass spectrometer (Applied Biosystems) was used [16].

### Promoter Studies and Microscopy

The promoter regions from −427 bp (*IPMI SSU1*), −725 bp (*IPMI SSU2*) and −1475 bp (*IPMI SSU3*) up to the translation start codon were amplified with the oligonucleotide pairs SSU1GUS.H/SSU1GUS.R (*IPMI SSU1*), SSU2GUS.H/SSU2GUS.R (*IPMI SSU2*) and SSU3GUS.H/SSU3GUS.R (*IPMI SSU3*). The PCR products were cloned into vector pBECKS19.101.1 using the restriction *Bam*HI and *Sal*I sites [17]. Constructs were checked by sequence analysis and used for plant transformation as described above. Tissue samples of at least three independent lines were investigated by histochemical GUS staining [18].

The *IPMI SSU1* gene including the promoter region was also fused to the reporter gene encoding the red fluorescent protein (RFP) from *Entacmaea quadricolor* (eqFP611) [19]. A PCR product covering the *IPMI SSU1* gene from position −432 to +753 (excluding the translation stop codon, positions given with respect to the ATG) was amplified with the oligonucleotide pair SSU1-RFP.H/SSU1-RFP.R and cloned via the *Bam*HI/*Sma*I restriction sites upstream of the RFP gene into vector peqFP611. The IPMI SSU1:RFP cassette was then transferred into the binary vector pMDC99 using the restriction sites *Bam*HI/*Eco*RI. This construct was used for transformation of *ipmi ssu2-1/ipmi ssu3-1* plants as described above.

Fluorescence microscopy was done either with an Axio Observer Z1 and the camera system AxioCam MRm (Carl Zeiss AG) using a MitoTracker filter set (HQ 545/30/HQ 610/75) [19] or the confocal microscope Leica TCS SP5 II with the laser DPSS 561 for excitation.

For light and electron microscopy, sections of different plant tissues were fixed in 2.5% glutaraldehyde (v/v), 1% sucrose (w/v) and 0.1 M MgCl_2_. The tissues were washed 3–4 times for 5–10 min in phosphate-buffered saline and post-fixed for 1–2 hours in 2% (w/v) aqueous osmium tetroxide. The samples were dehydrated in an ethanol series and infiltrated and embedded in EPON resin. Semi-thin sections (ca. 500 nm) and ultra-thin sections (ca. 80 nm) were cut with the microtome Ultracut (Reichert-Jung). Semi-thin sections were investigated using the microscope Axio Observer Z1 and the camera system AxioCam MRm (Carl Zeiss AG) whereas ultra-thin sections were investigated using the JEM-1400 electron microscope (JEOL, 120 kV) and the camera system Veleta (Olympus).

### Complementation Studies

Leu auxotrophic *E. coli* strains lacking intact *leuC* (CV522) and *leuD* (CV524) were obtained from the *E.coli* Genetic Stock Center (New Haven). For complementation, the *leuC* and *leuD* genes were amplified from *E.coli* DH5α and cloned into pUC19 (pleuC-leuD). The *E. coli* genes were then replaced by *Arabidopsis* cDNAs coding for the different IPMI subunits. Experimental details on the complementation studies were given in Protocol S3.

### Miscellaneous Methods

The chlorophyll content was measured in 20–60 mg of leaf tissue from 32 day-old plants. The material was frozen and ground in liquid nitrogen and chlorophyll was extracted by addition of 1 ml 80% (v/v) acetone and an incubation for 30 min on ice in the dark. The extract was cleared by centrifugation and extraction was repeated twice with 0.5 ml 80% (v/v) acetone without incubation. All supernatants were mixed and used for measurements. The chlorophyll concentration per fresh weight was calculated as described previously [20].

Leaf pigment composition was analyzed by thin-layer chromatography of 500 mg 14 (Col-0 wild type) or 21 day-old seedlings (amiR-SSU1-B). Plant material was frozen, ground in liquid nitrogen and extracted by addition of 0.5 ml 80% (v/v) acetone. The extract was cleared by centrifugation and the extraction was repeated by addition of 1 ml 80% (v/v) acetone and incubation for 30 min on ice in darkness. After clarification 40 µl of the mixed extracts was separated on hydrophobic TLC silica gel plates 60 F_254_ (Merck KGaA) with methanol:acetone:water (15∶5:1) as mobile phase.

Quantification of PCR products was done using AIDA Image Analyzer v3.12 according to instructions given by the manufacturer (Raytest, Germany).

## Results

### Knockdown of the IPMI SSU1 causes Severe Phenotypic Changes

**Figure 1:**
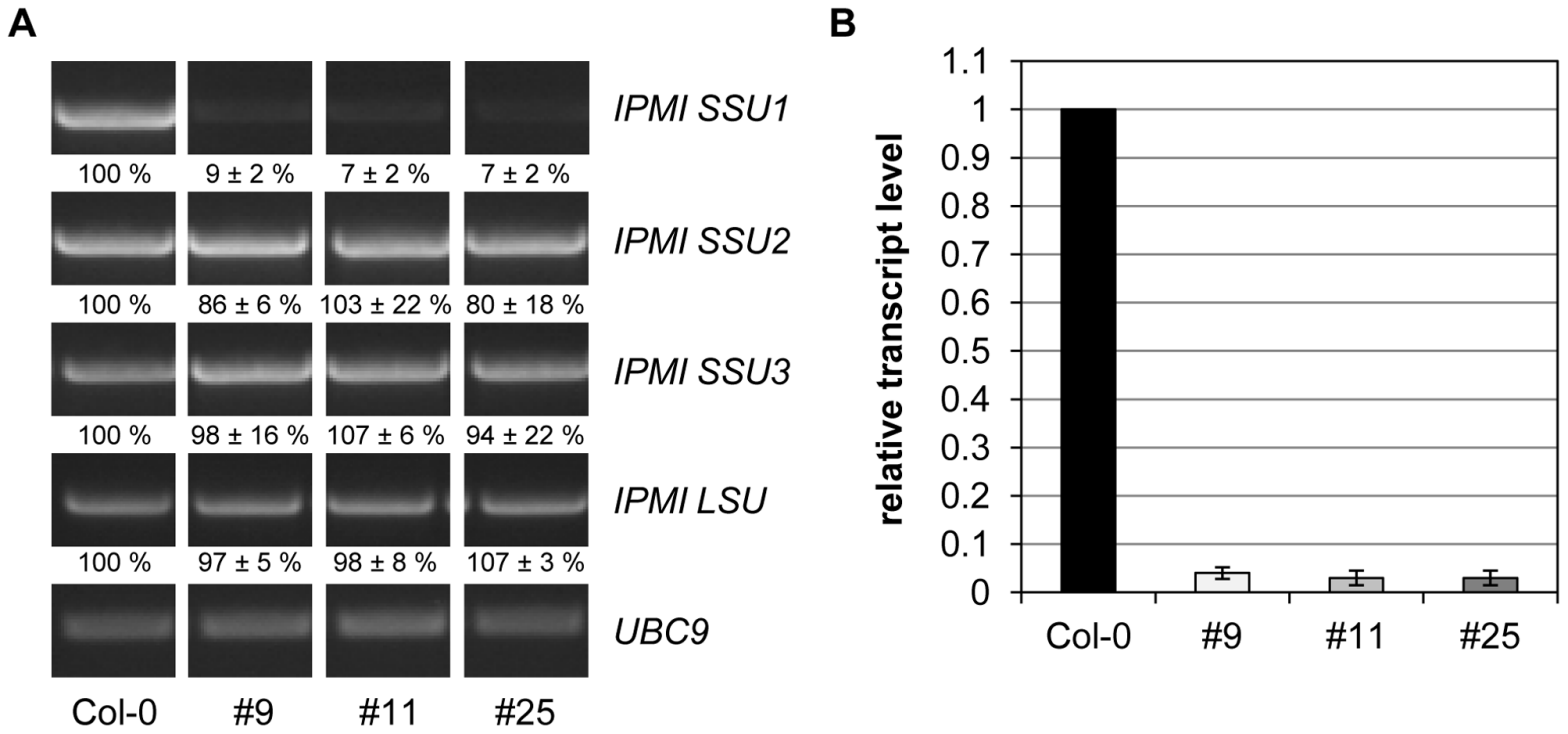
Investigation of *IPMI SSU* transcript levels. (A) Semi-quantitative RT-PCR analysis of Col-0 wild type and three different lines (#9, #11 and #25) expressing amiR-SSU1-B. This analysis was done with three technical and two biological replicates. Analyzed transcripts are indicated on the right hand side of the image. The input of equal amounts of cDNA was tested with a PCR detecting *UBC9* transcripts. Relative levels of the PCR products were normalized to *UBC9* mRNAs and are given below the images with respect to wild type. (B) Real Time qRT-PCR of IPMI SSU1 mRNA levels in the same knockdown lines.

Previous studies showed that an intact gene encoding the small subunit 1 of the isopropylmalate isomerase (*IPMI SSU1)* is essential for plant reproduction [3], [5], which prevented any study of the specific function of this gene *in vivo*. To overcome this problem, we cloned a construct to express an artificial microRNA specifically targeting the *IPMI SSU1* mRNA (amiR-SSU1-B, Fig. S1). After transformation of the amiR-SSU1-B construct into *Arabidopsis* Col-0 plants, several Basta-resistant transformants that showed aberrant morphological phenotypes were selected for detailed analyses (see below). To investigate the selective knockdown of *IPMI SSU1*, semi-quantitative RT-PCR was performed with three independent lines (amiR-SSU1-B #9, #11 and #25). This experiment revealed a striking reduction of the *IPMI SSU1* mRNA, while the transcript levels of the *IPMI SSU2*, *IPMI SSU3* and the *IPMI LSU1* genes were found to have undergone no significant changes (Fig. 1A). To measure the reduction of the *IPMI SSU1* mRNA level Real Time qRT-PCR was performed with the same amiR-SSU1-B lines demonstrating a strong knockdown of *IPMI SSU1* to less than 5% of the wild-type transcript level (Fig. 1B).

**Figure 2:**
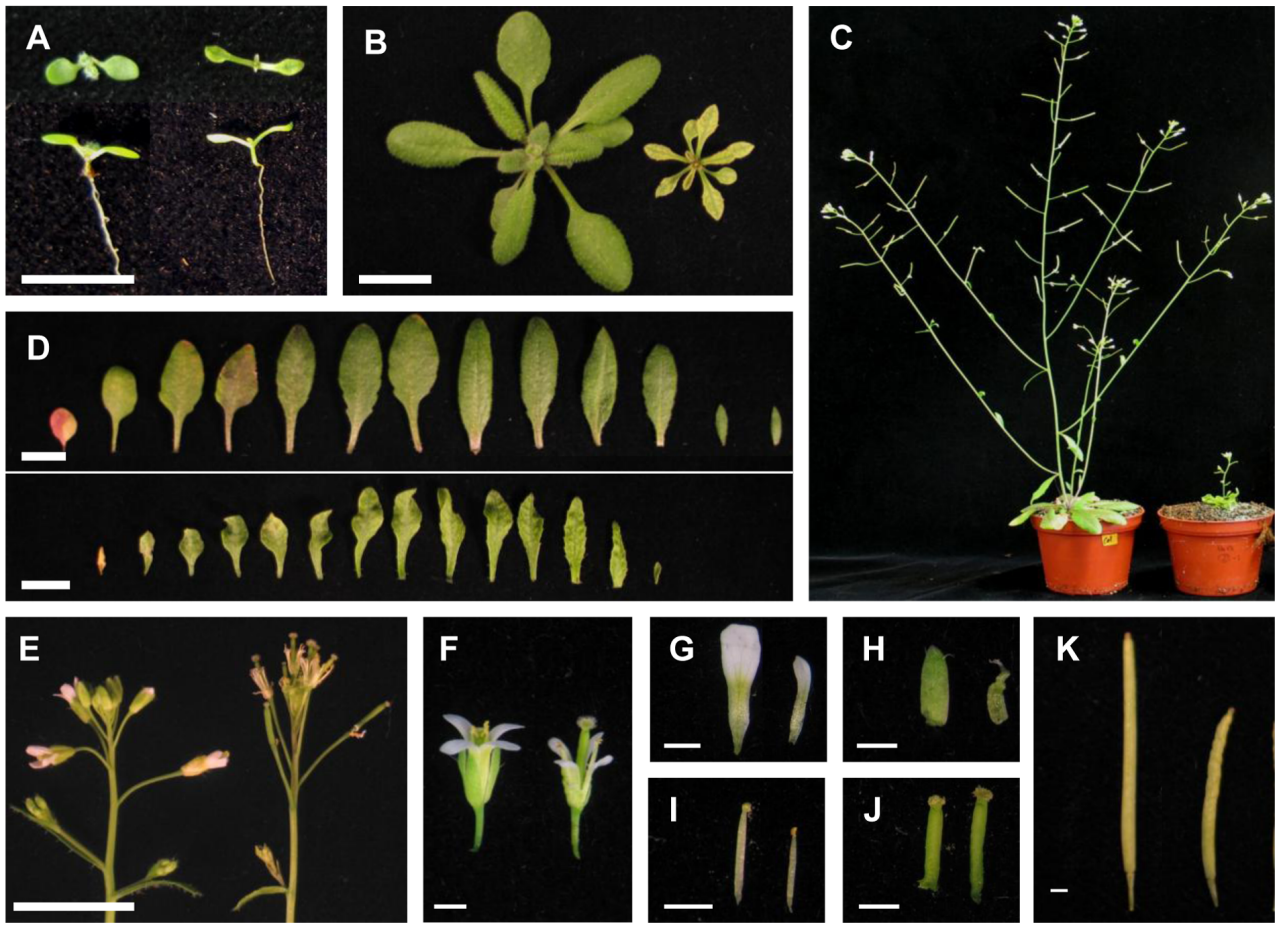
Macroscopic characterization of amiR-SSU1-B knockdown plants. In images (A) to (C) and (E) to (K) Col-0 wild type is shown on the left side while amiR-SSU1-B line #9 is on the right side. In image (D) Col-0 wild type is shown in the upper part, whereas amiR-SSU1-B line #9 is shown in the lower part. (A) to (C) show 8, 21 and 35 day-old plants, respectively. (D) Rosette leaves of 35 day-old plants. (E) Primary inflorescences, (F) flowers, (G) petals, (H) sepals, (I) stamen, (J) and gynecium of 42 day-old plants. (K) shows mature siliques. Thick scale bars correspond to 1 cm, thin scale bars to 0.1 cm.

As mentioned above the amiR-SSU1-B plants showed a severe macroscopic phenotype (Fig. 2A and B). They had narrow, often undulated leaves with yellow pale green leaf blades except at the vascular bundles, which showed normal green color (Fig. 2D). Germination and early phases of plant development were more or less indistinguishable from wild type, although cotyledons already showed the same phenotype as the leaves (Fig. 2A). About four or five days after germination the knockdown plants exhibited a severely retarded development resulting in a stunted growth. After four weeks the reduced growth resulted in plant fresh weights with less than 50% of wild type (Fig. S2A). AmiR-SSU1-B plants started to develop a small inflorescence about one or two weeks later than wild-type plants (Fig. 2C). Depending on the line, flowers showed an abnormal phenotype with stunted sepals, small narrow petals, smaller stamens and enlarged carpels. Seed pods were smaller and contained less seeds with normal size and germination rates (Fig. 2E to K). To confirm that the observed phenotypes are indeed linked to the knockdown of *IPMI SSU1* and to exclude off target effects, we established plants expressing two other amiRNA constructs (amiR-SSU1-C and D) targeting other sites in the *IPMI SSU1* mRNA (Fig. S1 and S3). Plants expressing these amiRNA showed very similar but even stronger phenotypes than the amiR-SSU1-B line. Growth of these plants was very slow, they generally remained very small and developed a bushy phenotype. Generally flowering started much later than in wild type and only very few fertile seeds were formed. Because of the severe phenotype of the amiR-SSU1-C and D plants we concentrated our further studies on plant lines expressing amiR-SSU1-B.

Taken together these results demonstrated that the knockdown of IPMI SSU1 leads to a severe macroscopic phenotype. This observation strongly suggests an important function of *IPMI SSU1* for normal growth and development particularly for the formation of leaves in *Arabidopsis*.

### IPMI SSU1 Knockdown Plants Contain Chloroplasts with Abnormal Morphology

**Figure 3:**
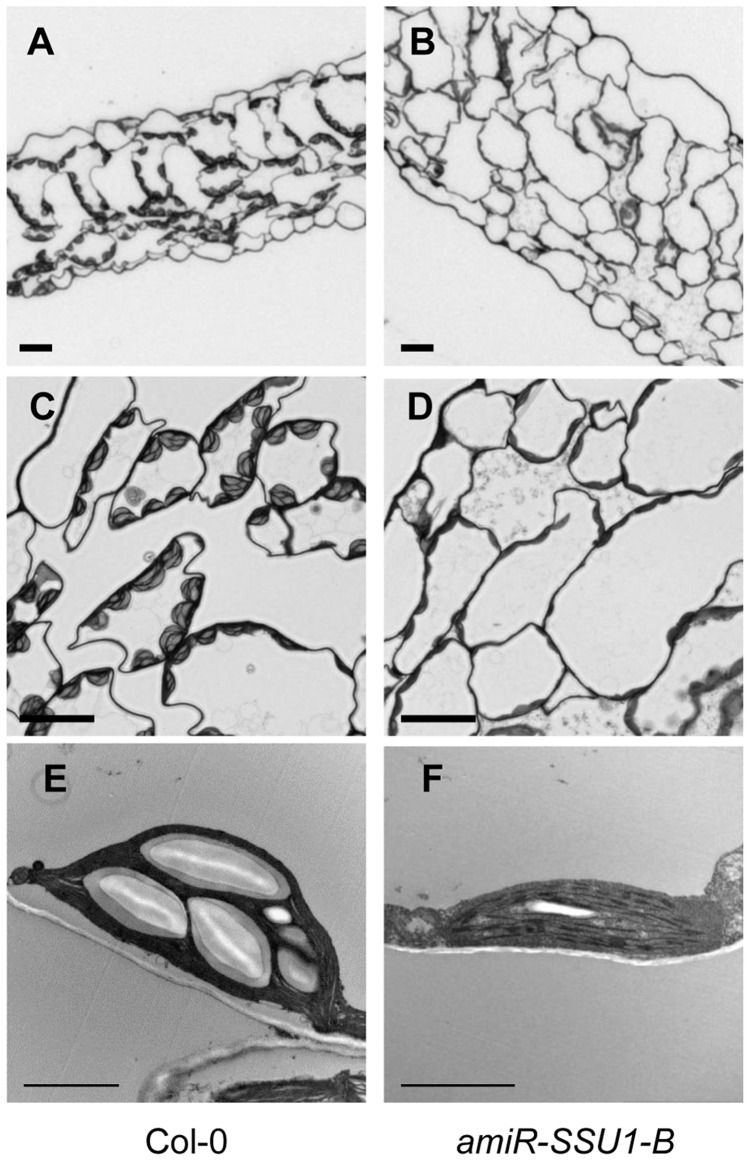
Microscopic examination of amiR-SSU1-B leaves. Images show cross sections through leaves of Col-0 wild type (A, C, E) and amiR-SSU1-B line #9 (B, D, F). Scale bars correspond to 20 µm (A to D), or 2 µm (E and F).

The interveinal leaf phenotype suggests that the knockdown of *IPMI SSU1* might also affect chloroplast development. To address this issue, cross sections of leaves were analyzed by light and transmission electron microscopy. In mutant leaves, chloroplasts have a shape different from the one in wild type (Fig. 3). Typically, these organelles from the mutant were flattened and contained less and smaller starch granules (Fig. 3E and F). Consistent with these observations and with the pale green color, the amiR-SSU1-B plants contained less than 50% of the chlorophyll in wild type (Fig. S2B). A comparison of the major leaf pigments did not reveal any difference in the composition of chlorophyll or carotenoids between the amiR-SSU1-B and wild-type plants (Fig. S2C). Despite the abnormal size and shape, the electron micrograph did not reveal clear changes of the thylakoid structure. In line with this observation an immunodetection analysis did not reveal any substantial differences in the levels of several chloroplast proteins (Fig. S2D). This includes both nuclear- and chloroplast-encoded proteins of photosystems I and II as well as chloroplast-encoded subunits of the cytochrome b_6_f complex and the ATP synthase. These results demonstrated that the strong knockdown of subunit 1 of IPMI results into a retarded growth, but no influence was seen on the accumulation of components of the major photosynthesis complexes.

Besides the abnormal chloroplast morphology, leaves of most amiR-SSU1-B plants showed an atypical patterning. In contrast to wild-type leaves, in which palisade cells could be easily discriminated from the spongy parenchyma cells, the mesophyll of amiR-SSU1-B leaves showed no apparent adaxial-abaxial patterning. Rather the whole mesophyll exhibited the characteristics of the spongy parenchyma. In addition, mutant leaves had more cell layers, which increased the thickness of the leaf along the adaxial-abaxial axis (Fig. 3A and B).

### Administration of Leu does not Restore the Interveinal Leaf Phenotype

**Figure 4:**
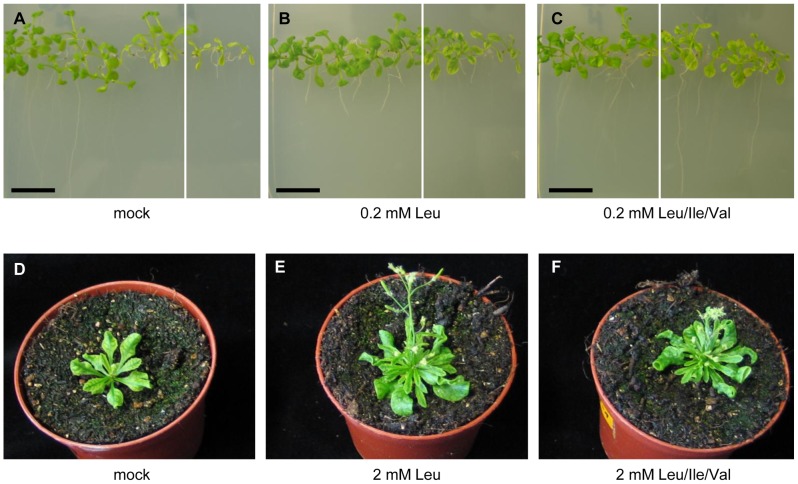
Supplementation experiments with branched-chain amino acids. (A to C) Col-0 wild-type (left parts of the images) and amiR-SSU1-B seedlings (line #9, right hand parts) were germinated and grown for 10 days on MS medium without exogenous amino acids (A, mock), in the presence of 0.2 mM Leu (B) and in the presence of 0.2 mM BCAA (C). (D to F) amiR-SSU1-B plants (line #9) were grown for 35 days on soil. Plants were watered without amino acids (D), with 2 mM Leu (E) or 2 mM BCAA (F).

One explanation for the severe macroscopic phenotype of amiR-SSU1-B plants is that the knockdown of *IPMI SSU1* expression simply caused a deprivation of Leu suggesting that administration of exogenous Leu would restore normal growth and development. To address this issue, amiR-SSU1-B knockdown plants were germinated and grown on MS medium in the presence of Leu, all three branched-chain amino acids (BCAA) or in the absence of exogenous amino acids. Both the addition of Leu and of all three BCAA stimulated growth of amiR-SSU1-B seedlings on MS-plates in comparison to the knockdown plants grown in the absence of exogenous amino acids. However, the administration of the amino acids did not restore normal leaf development in the knockdown plants (Fig. 4A to C). In wild-type plants, the addition of Leu and of all three BCAA inhibited growth of seedlings as observed previously [14], [21].

A similar observation was made after long term application of amino acids to plants grown on soil. Here supplementation of either Leu or the three BCAA stimulated growth but did not influence the morphological phenotype of the leaves (Fig. 4D to F). These data demonstrated that the observed interveinal and developmental leaf phenotype is not related to a simple reversible Leu deficiency.

### Metabolite Profiling Demonstrates the Function of *IPMI SSU1* Both in Leu and Glucosinolate Metabolism

To get a detailed picture about the function of *IPMI SSU1* we performed a metabolite profiling of amiR-SSU1-B and wild-type plants and seeds. In rosette leaves of amiR-SSU1-B plants, the content of total free amino acids was significantly decreased by a factor of 0.8 (Table 1). Significant reductions in the same range were seen for Asp, Gln, Glu, Gly, Ser and α-aminobutyrate, while the level of Ala was reduced by a factor of 0.5. In contrast, a moderate significant increase was seen for Asn (×1.4) and Met, while a clear elevation of Val (×4.6) was measured as expected upon a disruption of the pathway towards Leu. Surprisingly, we also measured an increase of Leu (×4.4), although the absolute levels of this amino acid are quite low (Table 1). Amino acid profiling of seeds revealed only three minor significant decreases of Asp, Trp and Tyr (×0.7 or 0.8) and a significant increase of S-methylmethionine (SMM) in the amiR-SSU1 knockdown line (Tables S2 and S4).

**Table 1:** Amino acid content in rosette leaves of amiR-SSU1-B plants.

Amino Acid	Amino Acid Content [nmol/mg Dry Weight]	
	Col-0	amiR-SSU1-B
Ala	4.8±0.5	2.5±0.5*
Arg	n.d.	0.1±0.1
Asn	3.5±0.4	4.7±1.0*
Asp	20.0±1.0	17.1±2.0*
Gln	85.0±8.9	69.3±10.0*
Glu	25.2±1.0	18.2±3.2*
Gly	4.3±0.2	2.7±0.7*
His	0.2±0.1	0.2±0.1
Ile	0.7±0.1	0.5±0.3
Leu	0.1±0.0	0.5±0.2*
Lys	0.2±0.0	0.2±0.0
Met	n.d.	0.3±0.1*
Phe	0.4±0.0	0.4±0.1
Ser	5.0±0.3	3.5±0.5*
SMM	0.1±0.1	0.1±0.1
Thr	1.7±0.1	1.8±0.2
Trp	n.d.	0.1±0.1
Val	1.2±0.1	5.5±1.6*
α-AB	1.9±0.3	1.4±0.2*
Total	152.3±11.8	127.7±15.5*

n.d., not detectable;

*p<0.01 in a statistical T-Test between Col-0 and amiR-SSU1-B.

Apart from amino acids, we also analyzed glucosinolates since many genes and proteins involved in BCAA metabolism have functions also in the biosynthesis of this class of secondary metabolites. In rosette leaves, we observed an increase of total glucosinolate content by a factor of 1.6 (Table 2). This includes almost all aliphatic glucosinolate species as well as the two indolic metabolites 1-methoxyindol-3-ylmethylglucosinolate (1MOI3M) and 4-methoxyindol-3-ylmethylglucosinolate (4MOI3M). In seeds, a decrease of almost all glucosinolate species added up into a slight decrease of the total content. The single exception was the increase of 5-methylsulfinylpentyl glucosinolate (5MSOP×10.9) while 5-methylthiopentyl glucosinolate was decreased (×0.6) (Table S3). Analogous changes were also observed in seeds of knockout mutants of *BCAT4*, whose encoded protein catalyzes the initial step of the Met chain elongation pathway [6].

**Table 2:** Glucosinolate profile of rosette leaves of amiR-SSU1-B plants.

Glucosinolate	Glucosinolate Content [µmol/g Dry Weight]	
	Col-0	amiR-SSU1-B
3MSOP	1.9±0.1	3.7±0.6*
4MSOB	15.1±0.9	25.9±2.5*
5MSOP	0.5±0.0	0.9±0.1*
7MSOH	0.4±0.0	0.5±0.1*
8MSOO	2.0±0.1	2.9±0.4*
4MTB	0.5±0.1	0.6±0.1
I3M	2.0±0.2	2.3±0.2
1MOI3M	0.5±0.1	0.7±0.1*
4MOI3M	0.3±0.0	0.4±0.1*
Total	23.1±1.5	37.9±3.2*

3MSOP, 3-methylsulfinylpropylglucosinolate; 4MSOB, 4-methylsulfinylbutylglucosinolate; 5MSOP, 5-methylsulfinylpentylglucosinolate; 7MSOH, 7-methylsulfinylheptylglucosinolate; 8-MSOO, 8-methylsulfinyloctylglucosinolate; 4MTB, 4-methylthiobutylglucosinolate; I3M, indol-3-ylmethylglucosinolate; 1MOI3M, 1-methoxyindol-3-ylmethylglucosinolate; 4MOI3M, 4-methoxyindol-3-ylmethylglucosinolate;

*p<0.01 in a statistical T-Test between Col-0 and amiR-SSU1-B.

We also searched for an accumulation of intermediates of both amino acid and glucosinolate biosyntheses by an LC-MS analysis of methanolic extracts collected during glucosinolate profiling. In leaves, we detected an accumulation of isopropylmalate (IPM) in amiR-SSU1-B plants, which demonstrated the role of IPMI SSU1 in Leu metabolism. We also found an accumulation of 2-(2′-methylsulfinyl)ethylmalate (2MSEM) and 2-(3′-methylsulfinyl)propylmalate (2MSPM) in the knockdown plants. 2MSEM and 2MSPM are oxidized derivatives of the native intermediates, 2-(2′-methylthio)ethylmalate and 2-(3′-methylthio)propylmalate, respectively, formed on plant extraction [5]. Although we could not make an absolute quantification of these glucosinolate intermediates, the peak areas indicated that both intermediates were found in substantially lower amounts in comparison to IPM (Table 3). None of these compounds was detected in wild-type leaves.

**Table 3:** Diverse metabolites in rosette leaves of amiR-SSU1-B plants.

Metabolite	Relative Content**	
	Col-0	amiR-SSU1-B
2-(2′-methylsulfinyl)ethylmalate	n.d.	307.3±115.5*
2-(3′-methylsulfinyl)propylmalate	n.d.	298.7±96.2*
flavonoid***	33339.8±2616.2	61926.0±7544.6*
isopropylmalate	n.d.	11512.7±1939.0*
sucrose	23050.9±3103.8	16860.8±3345.8*

n.d., not detectable;

*p<0.01 in a statistical T-Test between Col-0 and amiR-SSU1-B.

**peak area LC-IONTRAP-MS×1000,

***kaempferol-3-O-glucoside-7-O-rhamnoside.

In summary, the metabolite profiling data clearly demonstrated a dual function of IPMI SSU1. The considerable increase of Val and the accumulation of IPM in the IPMI SSU1 knockdown plants provide convincing evidence for the function of this protein in Leu biosynthesis, while the accumulation of 2MSEM and 2MSPM suggests a role of IPMI SSU1 in early cycles of Met chain elongation.

Besides the above mentioned changes we found a significantly decreased sucrose content in rosette leaves of amiR-SSU1-B plants (×0.7), while this metabolite was unchanged in seeds. In addition, the levels of different flavonoids were found to be increased in both leaves and seeds of the IPMI SSU1 knockdown plants. Interestingly also S-methylmethionine is enriched (×2.9) in seeds of the IPMI SSU1 knockdown mutant (Table 3 and Table S4).

### Changes in Phytohormone Levels

The metabolite profiling revealed an increase of both Leu and Val as well as total Met-derived aliphatic glucosinolates, although at the same time elevated levels of the corresponding precursor molecules were observed. This suggested that the knockdown of *IPMI SSU1* supports an increased biosynthesis of the two aliphatic amino acids and of Met-derived aliphatic glucosinolates. Since a previous study revealed an interference of the BCAA metabolism with the levels of several phytohormones [22], we measured the levels of jasmonate derivatives, salicylic acid and abscisic acid. While the latter signal molecule was significantly reduced by about 50%, significant increases were observed for jasmonate and its Ile conjugates (×4.1 to×5.2) as well as salicylic acid (×2.2) in leaves of the *IPMI SSU1* knockdown plants (Table 4).

**Table 4:** Phytohormone content in rosette leaves of amiR-SSU1-B and Col-0.

phytohormone	Phytohormone Content [ng/g Dry Weight]	
	Col-0	amiR-SSU1-B
salicylic acid	663.1±115.2	1435.8±471.8*
abscisic acid	52.4±16.8	25.7±8.0*
jasmonic acid	95.6±18.7	493.0±97.2*
(-)-jasmonic acid-L-Ile	1.2±0.7	5.7±1.1*
(+)−7-iso-jasmonic acid-L-Ile	3.2±1.4	13.2±3.2*
cis-OPDA**	1342.5±181.2	1148.3±332.7

*p<0.01 in a statistical T-Test between Col-0 and amiR-SSU1-B.

**cis-(+)−12-oxo-phytodienoic acid.

### Isopropylmalate Levels are Unchanged in *ipmi ssu2*/*ipmi ssu3* Double Knockout Mutants

To further define the individual functions of the three different small IPMI subunits we established an *ipmi ssu2-1*/*ipmi ssu3-1* double knockout by crossing previously established lines [5]. In contrast to amiR-SSU1-B knockdown plants the double knockout is phenotypically indistinguishable from wild type. The single mutants have different genetic background giving rise to distinct glucosinolate profiles in the parental accessions Ws (*ipmi ssu3-1*) and Col-0 (*ipmi ssu2-1*). Nevertheless we observed a complete lack of the C7 and C8 glucosinolates 7-methylsulfinylheptylglucosinolate (7MSOH) and 8-methylsulfinyloctylglucosinolate (8MSOO) in rosette leaves of these plants, while these glucosinolate species were detectable in each of the parental lines (Table S5). All C7 and C8 glucosinolates were also undetectable in seeds of the double knockout mutant (Table S6).

We also measured the contents of the above mentioned intermediates of Leu and glucosinolate biosyntheses. This was performed with total leaves, with samples enriched for the midrib and with leaf samples without midrib of Col-0 wild-type, of *ipmi ssu2-1*/*ipmi ssu3-1* double knockout and of amiR-SSU1-B plants. These assays were done with a more sensitive method that allowed a detection of IPM also in wild type. No significant differences were seen between IPM levels in wild type and in the *ipmi ssu2-1*/*ipmi ssu3-1* double knockout, while this compound was increased in all samples obtained from amiR-SSU1-B plants (Table 5). 2MSEM, an intermediate of the first round of Met chain elongation, was also more enriched in the *IPMI SSU1* knockdown plants than in the *ipmi ssu2-1*/*ipmi ssu3-1* double knockout plants, but was undetectable in wild type. When compared between the different tissues, this intermediate was lower in leaf samples without midribs from the *ipmi ssu2-1*/*ipmi ssu3-1* double knockout than in samples with midribs. A different pattern was seen for 2MSPM, an intermediate of the second round of Met chain elongation. This compound was enriched both in amiR-SSU1-B plants and in *ipmi ssu2-1/ipmi ssu3-1* double knockout mutant plants. It was found in highest amounts in total leaf and midrib samples of the *ipmi ssu2-1*/*ipmi ssu3-1* double knockout plants, and in levels 80% less in *IPMI SSU1* knockdown plants. In leaf samples without midribs, similar levels of 2MSPM were found in the double knockout and the amiR-SSU1-B knockdown plants. These data demonstrate that the knockdown of IPMI SSU1 alone triggers a stronger accumulation of 2MSEM than the knockout of both IPMI SSU2 and IPMI SSU3. Thus IPMI SSU1, which is involved in Leu biosynthesis, also takes part preferentially in the first cycle of Met chain elongation, and also in addition contributes to the second cycle. On the other hand, IPMI SSU2 and IPMI SSU3, which seem to have no role in Leu biosynthesis are involved in the second and later cycle of Met-derived glucosinolate chain elongation and may have a partial role in the first cycle, too.

**Table 5:** Accumulation of isopropylmalate and glucosinolate intermediates in different sections of rosette leaves of *ipmi ssu2-1*/*ipmi ssu3-1* and amiR-SSU1-B knockdown plants.

Metabolite	Relative content**		
	Col-0	*ipmi ssu2-1/ipmi ssu3-1*	amiR-SSU1-B
**Total leaf**			
2-(2′-methylsulfinyl)ethylmalate	n.d.	7.5±1.1§	12.7±3.4*
2-(3′-methylsulfinyl)propylmalate	1.1±0.1	204.9±31.1§	50.9±9.5*
isopropylmalate	41.7±8.8	36.1±5.9	8441.7±1032.8*
**Midrib**			
2-(2′-methylsulfinyl)ethylmalate	n.d.	7.9±4.9§	14.2±3.0*
2-(3′-methylsulfinyl)propylmalate	0.9±0.2	265.8±111.2§	76.8±12.0*
isopropylmalate	23.6±12.5	15.3±6.7	5123.8±1617.4*
**Leaf w/o midrib**			
2-(2′-methylsulfinyl)ethylmalate	n.d.	1.6±0.9§	14.0±1.7*
2-(3′-methylsulfinyl)propylmalate	0.7±0.2	50.3±30.4§	52.0±7.2*
isopropylmalate	15.4±3.8	19.3±6.4	8814.8±1839.8*

n.d., not detectable;

§ , p<0.01 in a statistical T-Test between Col-0 and *ssu2-1/ssu3-1*.

*p<0.01 in a statistical T-Test between Col-0 and amiR-SSU1-B.

**peak area LC-IonTrap-MS×1000.

In summary, these data demonstrate that IPMI SSU1 participates in both Leu and glucosinolate biosyntheses. Here it seems to be particularly important in the first cycle of the Met chain elongation. In contrast, IPMI SSU2 and IPMI SSU3 seem to have no substantial function in Leu metabolism under normal conditions. Moreover, these enzymes were found to be less important for the first step but essential for later cycles in Met chain elongation.

### Expression of the Different *IPMI SSU* Genes in *Arabidopsis*

**Figure 5:**
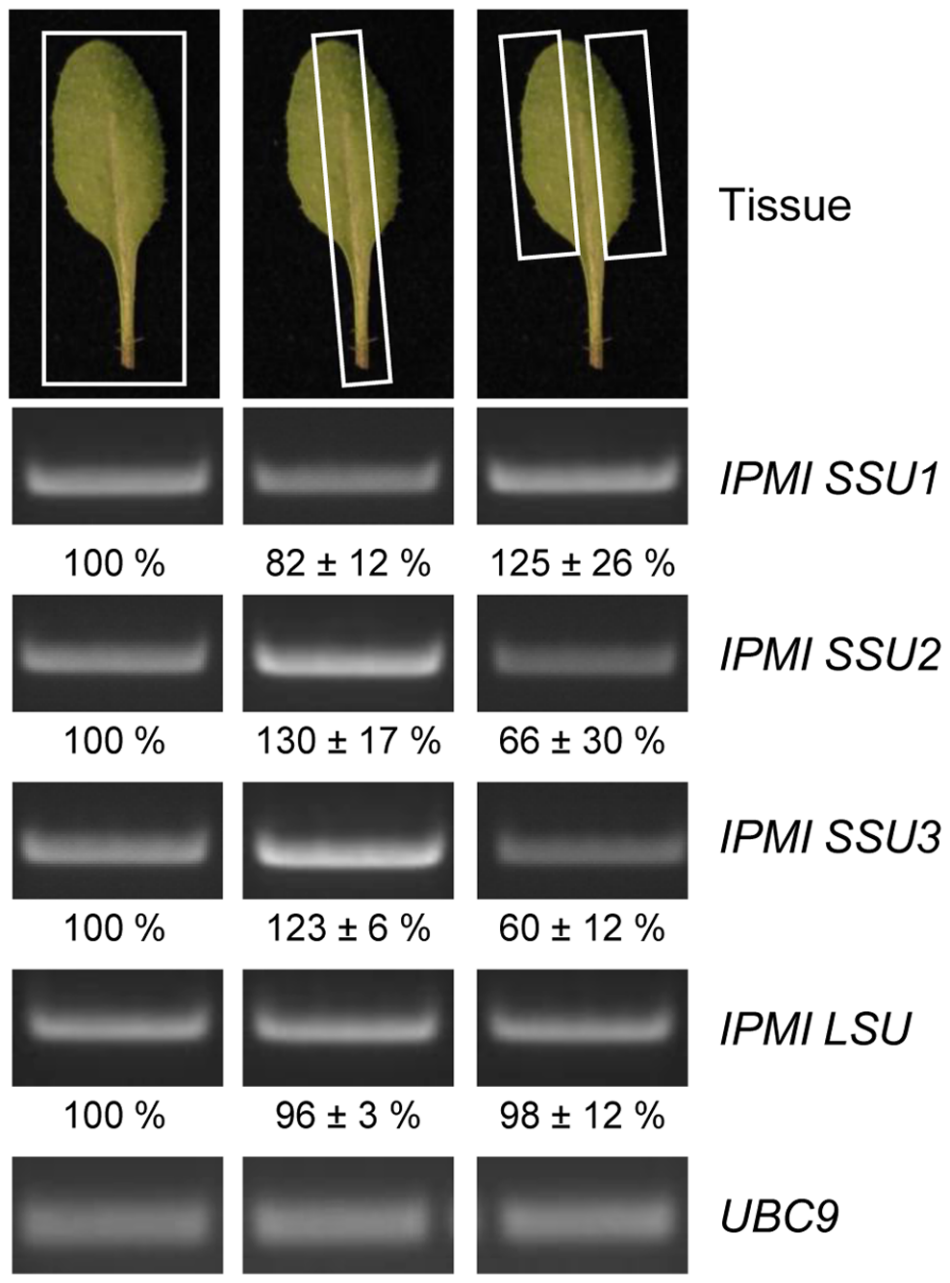
Semi-quantitative RT-PCR analysis of the *IPMI SSU* and *IPMI LSU1* mRNA levels. The transcripts of these genes were examined in different parts of the Col-0 wild-type leaves. Analyzed transcripts are indicated on the right hand side of the image. The use of equal amounts of cDNA was tested with *UBC9* transcripts. This analysis was done with three technical and three biological replicates. Relative levels of the PCR products are given below the images with respect to levels in whole leaves.

The differential accumulation of intermediates from Leu and glucosinolate biosynthesis in the different samples suggested a distinct tissue-specific expression pattern in leaves. Therefore we analyzed the transcript levels of the different IPMI SSU genes by semi-quantitative RT-PCR in total leaves, in samples enriched for the midrib and in leaf samples without midrib of Col-0 wild-type. Consistent with a predominant function of IPMI SSU2 and 3 in glucosinolate biosynthesis, transcripts of these genes were found to be enriched in midrib samples (130±13%; 123±6%), while they were less abundant in samples without this central leaf vein (66±30%; 60±12%), Fig. 5). On the contrary, transcripts of IPMI SSU1 were found to be less abundant in the midrib (82±12%) while they were slightly enriched in leaf samples without midribs (125±26%).

**Figure 6:**
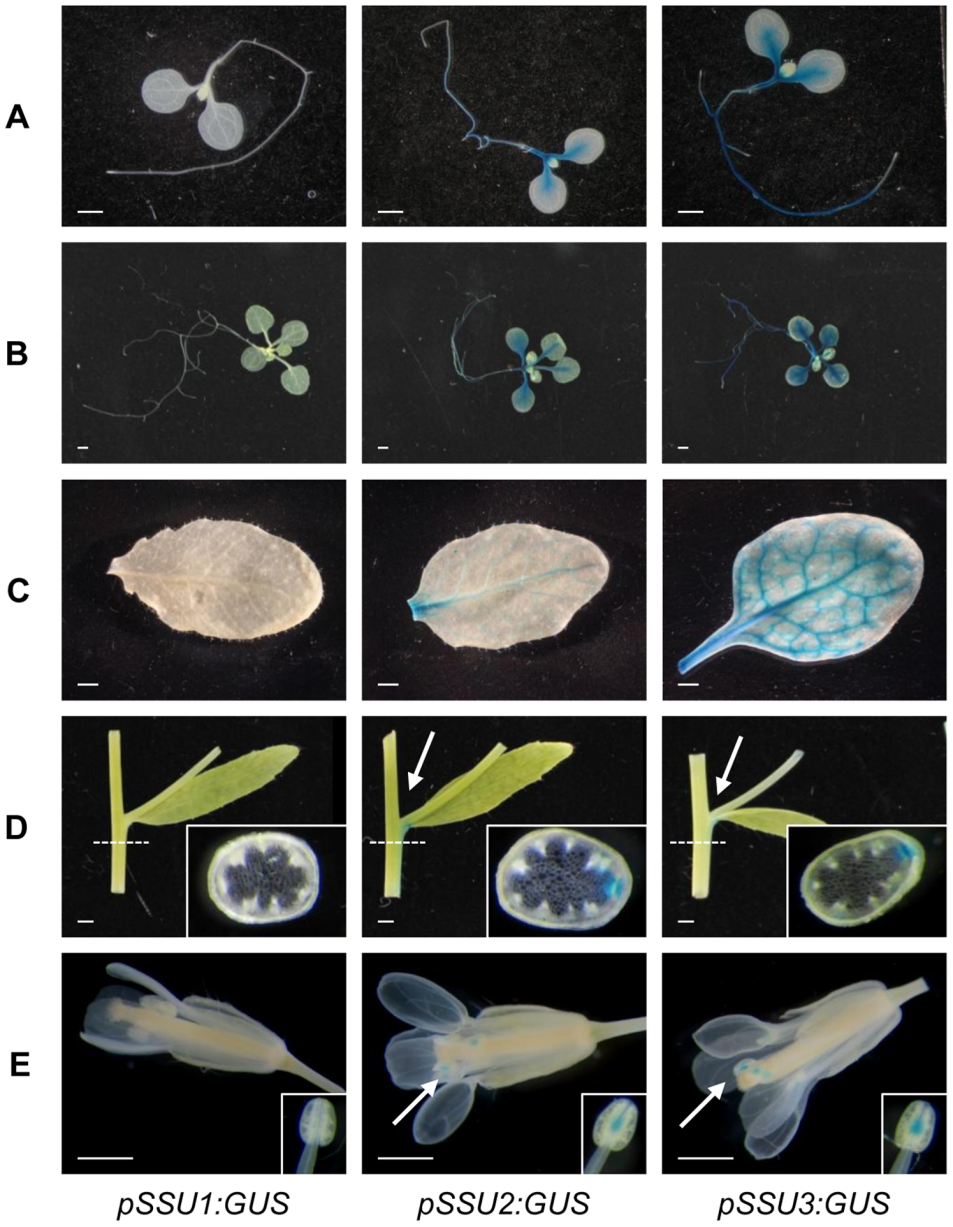
Histochemical GUS staining of plant tissues expressing the β-glucuronidase gene. The glucuronidase gene was expressed under the control of the IPMI SSU1 to 3 promoters (indicated in the top). (A and B) 8 and 15 day-old seedlings; (C) leaves, (D) sections and cross section (positions indicated by white dashed lines) of the inflorescence axis. White arrows indicate staining at the basis of branches of cauline leaves. (E) flowers, arrows indicate staining in the anthers, which are enlarged in the insets.

To substantiate these results we established promoter:glucuronidase (GUS) constructs for all *IPMI SSU* genes and established several independent lines for each construct. Consistent with the results of the semi quantitative RT-PCR histochemical GUS staining localized promoter activities of *IPMI SSU2* and *IPMI SSU3* to vascular bundles in roots and cotyledons of 8 or 15-day old seedlings and in rosette leaves of later stages (Fig. 6A to C). Promoter activity was also observed in stems of the inflorescence. Cross sections about 5 mm below a branch revealed an especially strong staining of the vascular bundle which branches off into a lateral inflorescence (Fig. 6D). These expression patterns were very similar to the one seen in a histochemical analysis of the IPMI LSU1 promoter [3]. In flowers, promoter activities of *IPMI SSU2* and *IPMI SSU3* were restricted to the connective tissue between the theca as was previously found for the *BCAT4* promoter (Fig. 6E) [23]. No GUS staining at all was observed for the *IPMI SSU1* promoter, which seems to be beyond the detection limit of the histochemical GUS assay (Fig. 6A to E). To overcome this problem, the complete *IPMI SSU1* gene (without translation stop codon) including a 432 bp promoter fragment was cloned upstream of the reporter gene encoding the red fluorescence protein eqFP611 (RFP) [19]. This reporter gene construct was stably integrated into the genome of the *ipmi ssu2-1*/*ipmi ssu3-1* double knockout mutant and several transformants were inspected by laser scanning microscopy. In these plants, weak fluorescence was observed in the root including the tip, at the basis of the hypocotyls and in emerging leaves (Fig. S4). However, the results of both reporter gene analyses consistently suggest only a comparatively weak activity of the *IPMI SSU1* promoter.

### Both Substrate Specificity and Tissue-specific Expression Determine Distinct Functions of IPMI in Glucosinolate Biosynthesis

The RT-PCR and promoter analyses of the different *IPMI SSU* genes suggests that tissue-specific expression might determine the functional specialization of the IPMI holoenzyme. But it might be also possible that other features of the different small subunits confer distinct functions of the holoenzyme. To investigate different influences we exchanged the promoters and reading frames between *IPMI SSU1* and *IPMI SSU3* genes and transformed the chimeric promoter:gene constructs into the *ipmi ssu2-1*/*ipmi ssu3-1* double knockout mutant. A glucosinolate profiling of seeds revealed that expression of both constructs did not induce the biosynthesis of C6 to C8 aliphatic glucosinolates (Table S6). Only a slight increase of 5-methylthiopentylglucosinolate (5MTP) was detectable. To the contrary, a specific increase of C3 glucosinolates (up to factor 10.6) was seen in seeds harvested from plants expressing the *IPMI SSU1* gene controlled by the *IPMI SSU3* promoter (*ipmi ssu2-1*/*ipmi ssu3-1+ pSSU3:SSU1)*. Moderate increases were found for C4 glucosinolate species with the highest enrichment seen for 4-methylthiobutylglucosinolate (4MTB, factor 2.5). In contrast to these plants, the expression of the *IPMI SSU3* gene controlled by the *IPMI SSU1* promoter resulted in an exclusive enrichment of C4 glucosinolate species, while C3 species were unchanged or even reduced. These results demonstrated that the *IPMI SSU3* promoter controlled expression of *IPMI SSU1* in tissues, where glucosinolates are usually synthesized, but triggered predominantly the biosynthesis of C3 glucosinolate species. Thus an IPMI holoenzyme containing IPMI SSU1 catalyzed almost exclusively the first round of Met-chain elongation although expression should also support synthesis of glucosinolate species with longer side chain. This observation strongly suggests that the substrate specificity of IPMI SSU1 is restricted to intermediates of the first and second cycle. In turn, ectopic expression of *IPMI SSU3* driven by the *IPMI SSU1* promoter results only in the biosynthesis of C4 glucosinolate species, suggesting that in this tissue environment, enzymes and intermediates for further elongation Met chain elongation cycles are absent. In addition, this result indicates that the *IPMI SSU1* promoter is active in tissues where the *IPMI SSU2* and *IPMI SSU3* promoters are shut down.

### Functional Studies of the IPMI Subunits by Complementation Studies using Leu Auxotrophic *E. coli* Strains

Our *in vivo* metabolite profiling described above and previous *in vivo* studies indicated that the large IPMI subunit and IPMI SSU1 are active in both Leu and glucosinolate metabolism, while IPMI SSU2 and 3 seem to act predominantly in Met chain elongation. To obtain information about the function of the *Arabidopsis* IPMI subunits with Leu, we performed complementation studies using two Leu auxotrophic *E. coli* strains, in which either the gene for the large subunit (strain CV522, Δ*leuC*) or the small subunit (strain CV524, Δ*leuD*) of the IPMI were inactive. Transformation of individual IPMI cDNAs from plants via the pUC19 vector into the different strains did not rescue Leu biosynthesis consistent with a previous report [3]. Thus we cloned *leuC* and *leuD* in a dicistronic context and exchanged the bacterial genes with plant cDNAs by trying to keep the intergenic region close to the original 10 bp sequence (Fig. S5).

**Figure 7:**
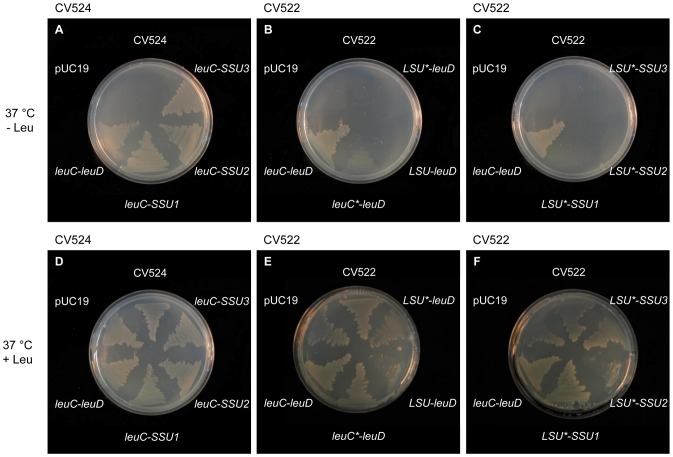
Complementation analysis of Leu-auxotrophic *E. coli* strains. Complementation capabilities of the different IPMI subunits were tested with the Leu-auxotrophic *E. coli* strains CV524 (Δ*leuD*, A, D) and CV522 (Δ*leuC*, B, C, E, F) on minimal medium with 1 mM IPTG and 2 mM Leu (D, E, F) at 37°C. The auxotrophic strains were transformed either with empty pUC19, *leuC*-*leuD*, or the different combinations of IPMI subunits *leuC*-*SSU1/2/3*, leuC*/*LSU*/LSU*-leuD or LSU*-*SSU1/2/3*. Untransformed bacteria were used as controls CV524 (Δ*leuD*) and CV522 (Δ*leuC*).

Introducing the different small subunits from *Arabidopsis* together with the original *leuC* from *E. coli* into the Δ*leuD* strain CV524 allowed the bacterial cells to grow on minimal medium in the absence of Leu (Fig. 7A). This result demonstrated that all small subunits can replace the function of the bacterial *leuD* gene in Leu biosynthesis as shown previously [3]. But contrary to this previous study, our result also showed that the small subunits from *Arabidopsis* are very well able to form functional heterodimers with the large subunit from *E. coli*.

We also tested whether the large IPMI subunit from *Arabidopsis* (*IPMI LSU1*) can restore Leu biosynthesis in strain CV522 lacking a functional *leuC* gene (Δ*leuC*). Therefore *leuC* was replaced by *IPMI LSU1* in the dicistronic construct, which was then transformed into CV522 cells. Under conditions identical to those used for complementation studies of the small IPMI subunits we never observed any growth on minimal medium in the absence of Leu (Fig. 7B). Thus the *Arabidopsis* gene encoding the large subunit of IPMI was not able to functionally replace *leuC* and thus could not restore Leu biosynthesis in strain CV522. To test whether this observation might be linked to the restriction site sequences introduced during the replacement of the *leuC* gene by the plant I*PMI LSU1* cDNA we replaced the *leuC* in a “natural” context by *leuC*, as we did for *IPMI LSU1*. The introduction of this construct (leuC*-leuD) into CV522 allowed only a severely retarded growth of the bacteria in the absence of Leu (Fig. 7B). This result demonstrated that changes in nucleotide sequences in close vicinity of the introduced reading frames, which are caused by the gene replacement, have a strong negative influence on ability of the *IPMI LSU* to restore Leu biosynthesis in CV522 (Fig. 7, upper, middle panel). However, we observed that growth of the CV522 cells with the reintroduced leuC* is much better at 21°C in comparison to 37°C, demonstrating an influence of the temperature on the complementation ability (Fig. S6).

Taken together the results of the complementation studies demonstrate that the small subunit from *Arabidopsis* can form heterodimers with the large subunit from *E. coli*, while the large subunit from the plant could not functionally replace the bacterial *leuC* in the dicistronic context.

## Discussion

### IPMI SSU1 Participates in Leu Biosynthesis and Early Cycles of the Met Chain Elongation Pathway

Previous studies showed the presence and general function of a bacterial-type heterodimeric isopropylmalate isomerase in *Arabidopsis thaliana*. While the large subunit of this enzyme (IPMI LSU1) operates both in Leu biosynthesis and Met chain elongation, there is a functional specification of the three small subunits (IPMI SSU1 to 3) [3], [5]. Our *in vivo* studies have now provided clear experimental evidence that IPMI SSU1 is crucial for Leu biosynthesis. This is supported by the enhanced accumulation of IPM and Val in the amiR-SSU1-B knockdown mutant. These plants also accumulated intermediates of glucosinolate formation (predominantly C3, less C4), which point to a substantial contribution of IPMI SSU1 to the first round of the Met chain elongation cycle as well. This function is confirmed by the predominant increase of C3 glucosinolate species upon the expression of the IPMI SSU1 gene under the control of the IPMI SSU3 promoter and the substantial amounts of C3 and C4 glucosinolate species in the *ipmi ssu2-1*/*ipmi ssu3-1* double knockout mutant (Table S5). In addition, the enrichment of Met in leaves and of SMM in seeds of amiR-SSU1-B plants supports the role of IPMI SSU1 in metabolism of Met-derived glucosinolates (Table 1, Tables S4–S6). Thus IPMI SSU1 has a dual function in Leu biosynthesis and the first cycle of the Met chain elongation pathway. On the contrary, our metabolite profiling of an *ipmi ssu2-1*/*ipmi ssu3-1* double knockout mutant showed the absolute requirement of these two genes for the biosynthesis of C7 and C8 glucosinolates, consistent with previous studies (Tables S5 and S6) [3], [5]. These subunits are dispensable for the synthesis of C3 and C4 glucosinolates, since these compounds are still present in substantial amounts in the double knockout mutant. Furthermore, IPMI SSU2 and SSU3 play no substantial role in Leu biosynthesis, since IPM does not accumulate to higher levels when both genes are inactivated. However, our current and previous complementation studies of *E. coli* Leu-auxotrophic strains revealed that all three small subunits from *Arabidopsis* (IPMI SSU1 to 3) can replace the function of the bacterial small subunit [3]. Thus IPMI SSU2 and/or 3 might contribute also to Leu biosynthesis in amiR-SSU1-B plants, probably in or along the vasculature, where the tissue has a normal green color (Fig. 2B). Although the amiR-SSU1-B plants exhibit a severe morphological phenotype, the metabolic phenotype was decisive in allowing a clear conclusion about the function of IPMI SSU1, as was true for the characterization of other lines mutated in genes of metabolism [5], [24]–[27].

Taken together, our data demonstrate that IPMI SSU1 has a major role in both Leu metabolism and in the early cycles of the Met chain elongation pathway. On the contrary, IPMI containing SSU2 or SSU3 subunits has a broad substrate range including Met derivatives with different side-chain lengths and IPM. In wild type plants, however, the conversion of IPM to Leu by IPMI SSU2 and/or IPMI SSU3 seems to play only a minor or even no role in the biosynthesis of Leu.

In contrast to previous studies our complementation showed the potential of the Arabidopsis derived IPMI SSU subunits to form functional heterodimers with the LeuC protein from *E. coli* [3]. In our experiments the *IPMI SSU* genes from *Arabidopsis* were co-transcribed with the *E. coli leuC* gene in a comparatively natural context. This gene arrangement allowed an expression of both genes similar to the situation in the genuine operon from *E. coli*. Such a co-transcription may be responsible for the complementation of Leu auxotrophy of strain CV524 by an IPMI composed of an *E. coli* large and *Arabidopsis* small subunit, which was not successful in an earlier study [3].

### The Knockdown of IPMI SSU1 Triggers Different Metabolite Changes in Leaves and Seeds

The knockdown of IPMI SSU1 differentially affects glucosinolate levels in leaves and seeds. The increased levels of glucosinolates in leaves are not reflected in seeds, where total glucosinolates were lowered by a factor of 0.8. All chain lengths decreased, and only 5MSOP and 8MTH increased or remained unaffected. At the onset of senescence, glucosinolates are transported from leaves to seeds [28], [29], so the drop of glucosinolates in seeds of amiR-SSU1-B plants may result from an interference of the IPMI SSU1 knockdown with the redistribution of glucosinolates during the senescence of the parental plant and the development of the seeds.

Fundamental differences between leaves and seeds were also observed for free amino acid. Amino acids are remobilized by protein degradation in source tissues such as mature leaves and are translocated into different sink tissues such as developing seeds and fruits [30]. This might be the reason for the increase of branched-chain and other amino acids in developing siliques [31]. Later, seed maturation is associated with the reduction of free amino acids (among many other metabolites) probably as a result of their use for biosynthesis of seed storage proteins [32]. Additionally, there might be amino acid biosynthesis in the embryo itself. Thus levels and composition of free amino acids in seeds are influenced at various stages by biosynthetic, catabolic, and reallocation processes, controlled by different regulatory circuits. This might explain the distinct differences in the content of certain amino acids in leaves and seeds with the latter being mostly unaffected by the knockdown of IPMI SSU1 (Table 1, Table S1). An exception is the non-proteinogenic amino acid SMM, the transport form of Met, which has previously been found to be enriched in plants impaired in Met chain elongation pathway [6], [14]. The increased amount of this compound in seeds of amiR-SSU1-B plants further supports the role of IPMI SSU1 in Met chain elongation (Table S4).

### Tissue-specific Expression Influences the Function of the Different Small IPMI Subunits

*IPMI SSU1* exhibits an expression pattern that is different from those of *IPMI SSU2* and *IPMI SSU3*. While the promoter activity of *IPMI SSU1* is below the detection limit in histochemical GUS assays, a fusion of the *IPMI SSU1* gene and its promoter to the RFP reporter gene revealed expression in multiple tissues (Figure S4). Moreover the *IPMI SSU1* transcript was easily detected by RT-PCR in this and a previous study [3], suggesting a comparatively high accumulation of this RNA potentially due to an increased stability. In addition, peptides of this protein have been detected in various tissues indicating that the protein also accumulates to relatively high levels despite the low promoter activity (http://fgcz-pep2pro.uzh.ch). The gene expression pattern is in contrast to that of *IPMI LSU1* that exhibits also a dual function in both Leu and glucosinolate biosyntheses. The promoter of *IPMI LSU1* showed relatively high activity mainly in or along the vasculature, similar to the promoters of *IPMI SSU2* and *IPMI SSU3*, whose gene products function mainly in glucosinolate biosynthesis [3]. The expression pattern of *IPMI LSU1*, *SSU2* and *SSU3* is consistent with the generally accepted concept that glucosinolates are formed in or immediately adjacent to the vascular system [33]. However, our results with *IPMI SSU1* suggest that the first cycle of Met chain elongation also occurs in other tissues. The introduction of the IPMI SSU3 reading frame under the control of the IPMI SSU1 promoter triggered an enhanced accumulation of C4 glucosinolates, while 5MTP is only marginally increased and C6 to C8 glucosinolates were undetectable (Table S6). By contrast, previous work and our studies show that the IPMI SSU3 protein expressed under its own promoter is associated with the formation of C7 and C8 glucosinolate species (Table S5) [3]. These observations can be resolved by assuming that the reactions forming Met derivatives with one or two additional methylene groups take place in a variety of tissues, while those forming longer Met derivatives are restricted to vascular tissues. Thus IPMI SSU1, which is expressed in multiple tissues (Fig. 6) uses 2MSEM and 2MSPM as substrates as part of the IPMI holoenzyme complex (Table 3) and participates in the formation of side chains for C3 and C4 glucosinolates. IPMI SSU3 can catalyze reactions in the formation of C7 and C8 glucosinolates, but when *IPMI SSU3* expression is driven by the *IPMI SSU1* promoter, only C4 glucosinolates are formed because the resulting holoenzyme only has access to 2MSEM and 2MSPM as substrates despite its capacity for catalysis of the IPM isomerase reaction in formation of Met derivatives with longer side chains.

These observations can also explain the glucosinolate phenotype of the different IPMI LSU1 knockdown mutants. In leaves of these lines, which contain the T-DNA insertions in the promoter regions, mainly C3 glucosinolate species accumulate, whereas almost all other glucosinolate species are reduced or even undetectable as for instance C7 and C8 species [5]. Since the T-DNA insertions cannot interfere with the substrate specificity of the IPMI, they apparently alter the expression pattern of the IPMI LSU1 gene. Considering the results described above it seems likely that the T-DNAs interfere with the expression of *IPMI LSU1* gene in the vasculature.

Considering the expression and substrate specificity of the other enzymes of Met chain elongation, it seems likely that in the first round of elongation methylalkylmalate synthase 1 (MAM1) delivers 2-(2′-methylthio)ethylmalate to IPMI LSU1 and SSU1. The isomerized product, 3-(2′-methylthio)ethylmalate, is further metabolized by one of two isopropylmalate dehydrogenases (IPMDH2 and/or 3) [34]–[39].

### Full Function of IPMI SSU1 is Essential for ad−/abaxial Patterning of Leaves

IPMDH2 and 3 are both also involved in Leu biosynthesis, with IPMDH3 contributing the majority of total cellular isopropylmalate dehydrogenase activity. IPMDH1 is predominantly involved in glucosinolate biosynthesis [24], [35], [39]. While single mutants of these genes are macroscopically indistinguishable from wild type, an *ipmdh2*/*ipmdh3* double knockout is lethal. But mutants containing only a single intact *IPMDH2* allele exhibit a mild interveinal chlorosis of leaves and reduced growth, a phenotype that is even stronger when IPMDH1 is knocked out. This phenotype is reminiscent of the morphology of the amiR-SSU1-B knockdown plants. However, the latter exhibit a much stronger form of this phenotype accompanied by additional characteristics like narrow cotyledons and leaves (Fig. 2). Additionally, the amiR-SSU1-B silencing mutants contain small almost starch-less chloroplasts, deformed flower organs and exhibit an aberrant adaxial-abaxial patterning of leaves, abnormalities not observed in the *ipmdh* mutants. The latter is manifested in a leaf mesophyll that lacks the characteristics of the palisade parenchyma (Fig. 3). Presently, it is unclear how the knockdown of the *IPMI SSU1* gene interferes with establishment of adaxial-abaxial polarity within the mesophyll. The formation and maintenance of organ polarity depends on sustained positional information provided by the meristem. A number of different but also partially redundant pathways are involved in this differentiation process including several adaxial and abaxial determinants, whose mutual relationships are important for precise adaxial and abaxial domains [40]. Defects in the adaxial determinants lead to a loss of dorsal cell types, similar to what is observed in leaves of amiR-SSU1-B plants suggesting that IPMI SSU1 gene function interferes with the function of such adaxial determinants. This relationship might be indirect involving various regulatory networks such as protein synthesis pathways. The latter is implicated by the abnormal dorsoventral leaf polarity found in mutants affecting different components of the cytosolic ribosomes [41]–[43]. Likewise, impaired function of the EMBRYO DEFECTIVE DEVELOPMENT1 gene, encoding a plastid- and mitochondrial-located glycyl-tRNA synthetase, leads to changes in the patterning in different parts of the leaf [44]. Thus both cytosolic and organellar protein syntheses are linked to organ polarity.

However, the level of Leu is not reduced but even slightly increased in knockdown amiR-SSU1-B plants and this curious result seems to exclude the simple explanation that severely impaired IPMI SSU1 gene function leads to a shortage in the supply of Leu for protein synthesis. But there are different subcellular compartments with their own free amino acid pools and it is possible that Leu is increased in a compartment where it is not readily available for transport to the cytosol or various organelles. This assumption is supported by the observation that administration of Leu supports enhanced growth of the *IPMI SSU1* silencing plants suggesting that there is indeed a critical shortage in Leu that can be partially compensated by the addition of exogenously added Leu. But the administered Leu (or BCAA) does not support growth as it is observed in wild type on soil and it does not compensate for the chlorotic phenotype of leaves and cotyledons, similar to what has been observed for comparatively weak phenotypes linked to impaired function of genes related to Leu biosynthesis [35], [45]. Thus defects in Leu biosynthesis lead to phenotypes that cannot be overcome by an exogenous supplementation of Leu or other BCAA as it has been observed in studies of other mutants in which for example a defect in the *HPA1* gene encoding histidinol-phosphate aminotransferase or the partial deficiency of *OMR1*, encoding threonine deaminase can be fully compensated by the administration of His and Ile, respectively [46], [47].

### Is the Chlorotic, Interveinal Phenotype a Result of an Imbalanced Distribution of Leu?

Besides a potential imbalance of the Leu concentrations within cells there might be also an uneven distribution of Leu within the leaf and other parts of the plants, which might also explain the phenotype of the amiR-SSU1-B plants. As indicated by the normal green color a residual Leu biosynthesis occurs in or along the vasculature by the participation of IPMI SSU2 and/or IPMI SSU3. But it seems that the *de novo* synthesized Leu cannot efficiently penetrate into the mesophyll, where this amino acid might be more or less absent due to the severe knockdown of IPMI SSU1. Narrow leaves of amiR-SSU1-B plants contain a comparatively high proportion of vascular tissue, which might explain the slight increase of Leu in total leaf tissue. But if this explanation is true, the administration of Leu should mainly support additional growth in and along the vasculature and its neighboring tissue, since the interveinal phenotype remains more or less unaffected by the additional growth.

The overrepresentation of vasculature in total leaf tissue might also explain the increased level of glucosinolates in the amiR-SSU1-B plants. Furthermore, the biosynthesis of these secondary metabolites might additionally be stimulated by the increased levels of jasmonates and partially also by the elevated levels of salicylic acid [2], [48]. On the contrary, lowered jasmonate levels have been observed in recent study of IPMDH double and partial triple mutants that show a similar phenotype as amiR-SSU1-B plants [49]. However, these plants show also a number of other striking differences to the amiR-SSU1-B knockdown. As mentioned above the macroscopic phenotype is less severe i.e. the plants do not exhibit narrow leaf blades or abnormal leaf patterning, and there has not been any anomalous formation of flower organs. But most importantly these plants have reduced levels of free Leu, a phenomenon that has never been observed in our measurements in amiR-SSU1-B plants (Table 1 and Table S2).

### Is Leu a Signaling Molecule in Plants?

Besides its function as building block for protein synthesis, a role of Leu as a signaling molecule is well established in animals. Here Leu regulates food intake, protein synthesis and autophagy, functions that involve TOR and S6K1 signaling pathways [50]–[53]. The presence of a number of components of these signaling cascades [54], [55] and a comprehensive combined metabolite and transcript profiling study suggest that Leu might also function as an endogenous signal in photosynthetic organisms [56]. Administration of predominantly Leu but to a lesser extent also of Val leads to retardation or even complete arrest of plant growth [14], [21]. This might at least in part be due to feedback inhibition of acetohydroxyacid synthase and isopropylmalate synthase by these amino acids [21], [57]. Possibly this phenomenon disguises other effects of Leu in plants, particularly its function as signal. In *Arabidopsis* the downregulation of the target of rapamycin (TOR) kinase leads to a decrease of photosynthesis, to an inhibition or arrest of growth, an increase of flavonoids and Trp- and Met-derived glucosinolates and a decrease of sucrose similar to what have been observed in amiR-SSU1-B plants [58]–[60]. Similarities were also observed with respect to Val and Leu, which were increased in both TOR and IPMI SSU1 knockdown plants. However, TOR downregulation triggers increased starch accumulation as well as elevated levels of many amino acids and this is in clear contrast to amiR-SSU1-B plants, which contain less starch and also decreased levels of amino acids (Fig. 3, Table 3). Therefore, it is rather unlikely that reduced growth induced by the knockdown of IPMI SSU1 is related to altered TOR activity although Leu levels are altered in amiR-SSU1-B plants. But as discussed above there might be an imbalance of Leu levels in leaf tissues which might have distinct effects on TOR activity. Thus it remains unclear whether Leu (or Val) have any influence on TOR activity in plants, in which the potential of amino acids as signaling molecules controlling gene expression has been suggested [56], [61]. Up to now sugars rather than amino acids have been indicated in signaling of the nutrient status in plants [54], [62].

## Supporting Information

Figure S1 Overview of the different amiRNA targeting *IPMI SSU1* in *Arabidopsis*. (A) Approximate target regions of the various amiR-SSU1 are indicated by arrows. White boxes indicate untranslated regions, a black box defines the coding region and the line represents an intron (B). Names and sequences of the amiRNA. The exact target regions are given with respect to ATG (A = +1). Bold letters represent the tenth position of each amiR-SSU1, underlined letters indicate nucleotides that mismatch with the target.
(TIF)

Figure S2 Characterization of amiR-SSU1-B plants. Comparison of fresh weight (A), chlorophyll content (B), leaf pigments composition (C) and levels of plastid proteins between amiR-SSU1-B and wild-type plants (D).
(TIF)

Figure S3 Phenotype of amiR-SSU1-C and -D plants. Macroscopic phenotype of about 35 day-old amiR-SSU1-C and D plants. White bars correspond to 1 cm.
(TIF)

Figure S4 Expression studies of the *IPMI* SSU1 gene. The complete gene (w/o translation stop codon) including a 432 bp promoter fragment was cloned upstream of the with the *RFP* gene (details see Material and Methods) and stably integrated into the genome of the *ipmi ssu2-1*/*ipmi ssu3-1* double knockout mutant. Confocal images showed red fluorescence in the following tissues: root (A), root tip (B), basis of hypocotyl (C), and emerging first leaf pair of 5 or 6 day-old seedlings (D). Thick scale bars correspond to 100 µm, thin scale bars to 20 µm.
(TIF)

Figure S5 Schematic overview of the constructs used for CV522 (Δ*leuC*) and CV524 (Δ*leuD*) for complementation studies. The dicistronic *leuC*-*leuD* was amplified from the leu operon (*leuABCD*) and cloned into pUC19. Afterwards *leuC* was exchanged by the cDNAs of *IPMI LSU1* from *Arabidopsis* with (LSU) or without 62 amino acids at the N-terminus, corresponding to the predicted plastid targeting sequence (LSU*). E. coli leuC* was cloned in the same way like *LSU* and LSU*. *leuD* was replaced by one of the *A. thaliana IPMI SSU1, 2* or *3*. Positions and orientations of the primers used for PCR are indicated by bent arrows. The following oligonucleotides have been used: A, leuC-comp.H; B, leuD-comp.R; C, LeuCErsatz1 AscI; D, LeuCErsatz2 PacI; E.1, IPMILSU1AscI; E.2, LSU-Chlp.target.AscI; F, IPMILSU2PacI; G, LeuC_AscI; H, LeuC_PacI; I, LeuDErsatz1 AscI; K, LeuDErsatz2 PacI; L.1, IPMISSU1.1AscI; L.2, IPMISSU2.1AscI; L.3, IPMISSU3.1AscI; M.1, IPMISSU1.2PacI; M.2, IPMISSU2.2PacI; M.3, IPMISSU3.2PacI; N, LSU-Xba; O, LeuDErsatz2 SmaI; P.1, IPMISSU1.XbaI; P.2, IPMISSU2.XbaI; P.3, IPMISSU3.XbaI; Q.1, IPMISSU1.SmaI; Q.2, IPMISSU2.SmaI; Q.3, IPMISSU3.SmaI. Oligonucleotide sequences are given in Table S1.
(TIF)

Figure S6 Complementation analysis of CV522 (Δ*leuC*) at 22°C. Complementation capability of the different IPMI subunits in the Leu-auxotrophic *E. coli* strain CV522 (Δ*leuC*, A, B) was tested on minimal medium with 1 mM IPTG at 21°C. The auxotrophic strain was transformed either with empty pUC19, *leuC*-*leuD,* or with different combinations of IPMI subunits, leuC*/*LSU*/LSU*-*leuD* (A) and LSU*-*SSU1/2/3* (B).
(TIF)

Table S1 Oligonucleotide sequences.
(PDF)

Table S2 Amino acid profile in seeds of amiR-SSU1-B plants.
(PDF)

Table S3 Glucosinolate profile in seeds of amiR-SSU1-B plants.
(PDF)

Table S4 Relative content of diverse metabolites in seeds of amiR-SSU1-B plants.
(PDF)

Table S5 Glucosinolate profile in rosette leaves of an *ipmi ssu2-1*/*ipmi ssu3-1* double knockout mutant.
(PDF)

Table S6 Glucosinolate content in seeds of pSSU1:SSU3 and pSSU3:SSU1 lines.
(PDF)

Protocol S1 Metabolite analysis by LC-MS/MS.
(DOCX)

Protocol S2 Analysis of isopropylmalate and intermediates of glucosinolate biosynthesis.
(DOCX)

Protocol S3 Complementation studies leucine auxotrophic *E. coli* strains.
(DOCX)
