# Supplementary figures and images for: The Small Subunit 1 of the Arabidopsis Isopropylmalate Isomerase Is Required for Normal Growth and Development and the Early Stages of Glucosinolate Formation
Source: PLoS One. 2014 Mar 7;9(3):e91071. doi: 10.1371/journal.pone.0091071 (PMC3946710; doi:10.1371/journal.pone.0091071)

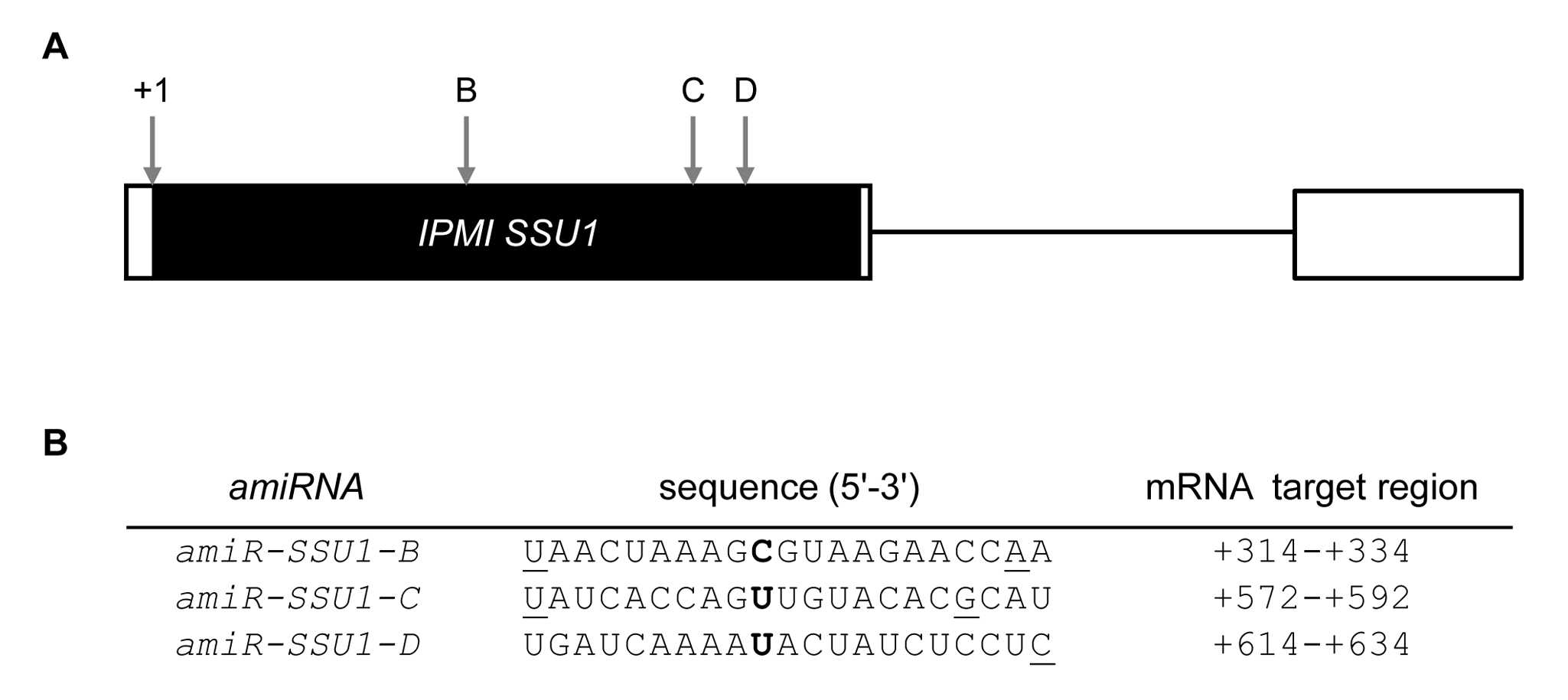

Supplement: Figure S1 — Overview of the different amiRNA targeting IPMI SSU1 in Arabidopsis. (A) Approximate target regions of the various amiR-SSU1 are indicated by arrows. White boxes indicate untranslated regions, a black box defines the coding region and the line represents an intron (B). Names and sequences of the amiRNA. The exact target regions are given with respect to ATG (A = +1). Bold letters represent the tenth position of each amiR-SSU1, underlined letters indicate nucleotides that mismatch with the target. (TIF) [file pone.0091071.s001.tif]

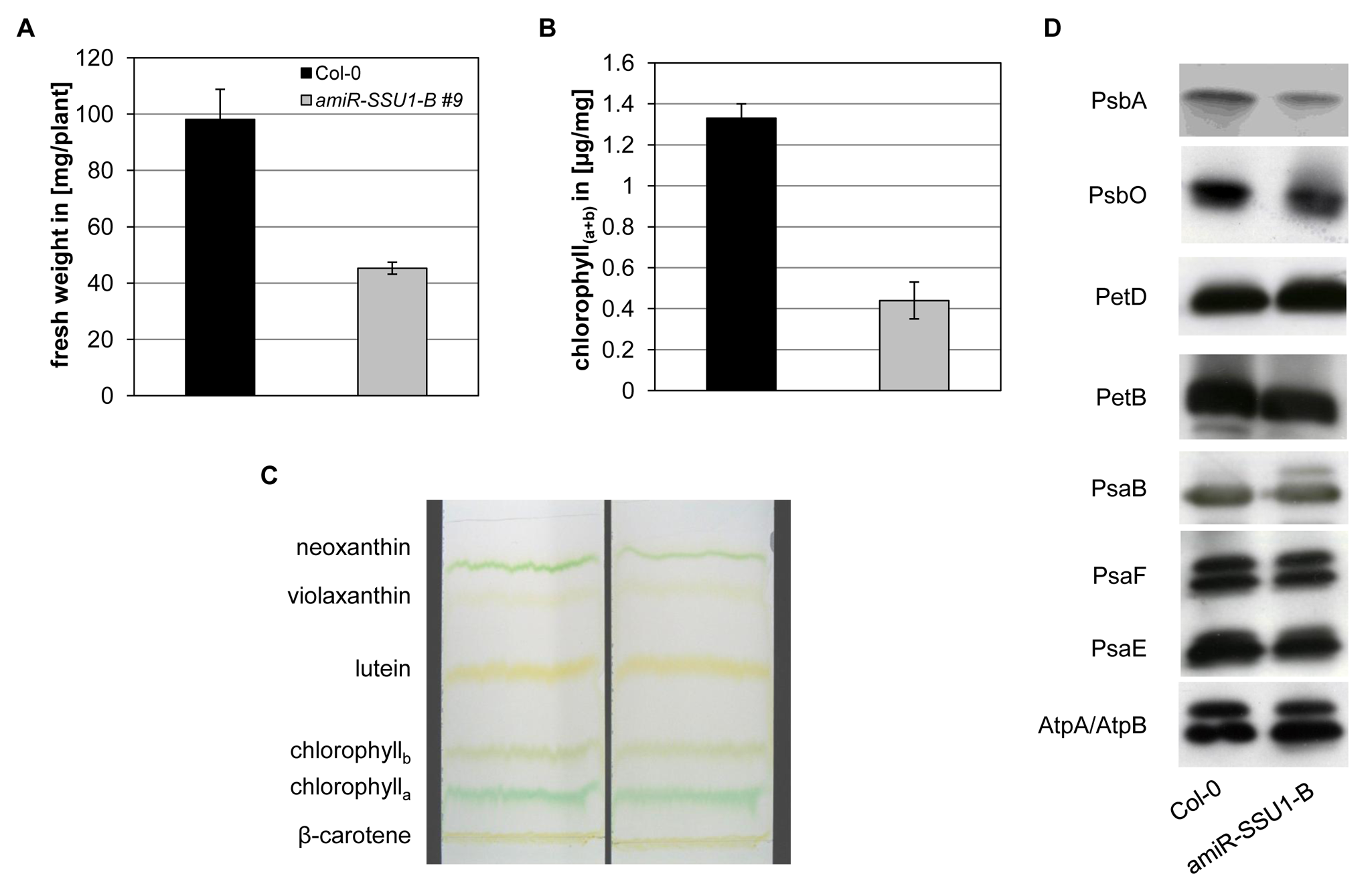

Supplement: Figure S2 — Characterization of amiR-SSU1-B plants. Comparison of fresh weight (A), chlorophyll content (B), leaf pigments composition (C) and levels of plastid proteins between amiR-SSU1-B and wild-type plants (D). (TIF) [file pone.0091071.s002.tif]

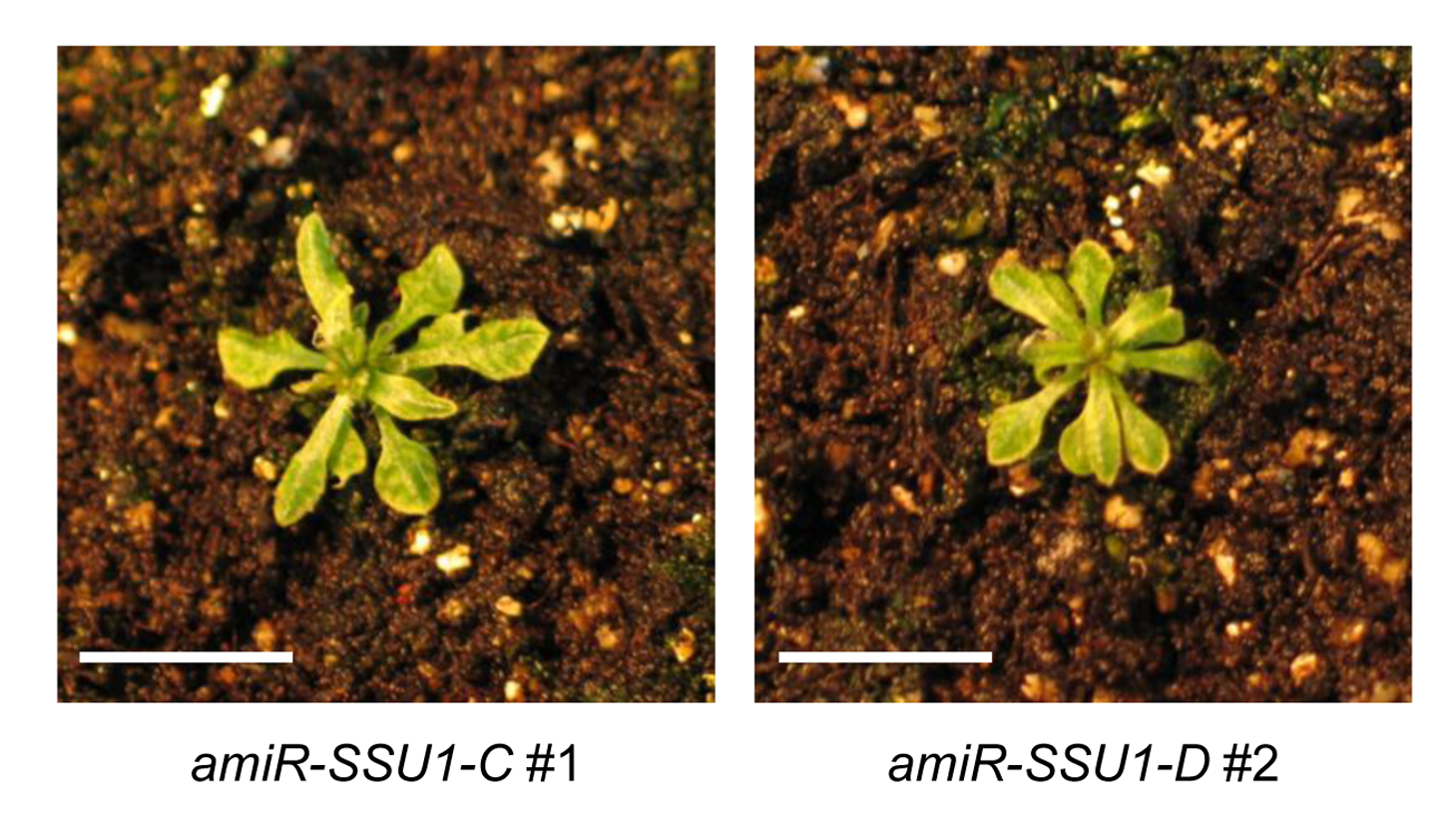

Supplement: Figure S3 — Phenotype of amiR-SSU1-C and -D plants. Macroscopic phenotype of about 35 day-old amiR-SSU1-C and D plants. White bars correspond to 1 cm. (TIF) [file pone.0091071.s003.tif]

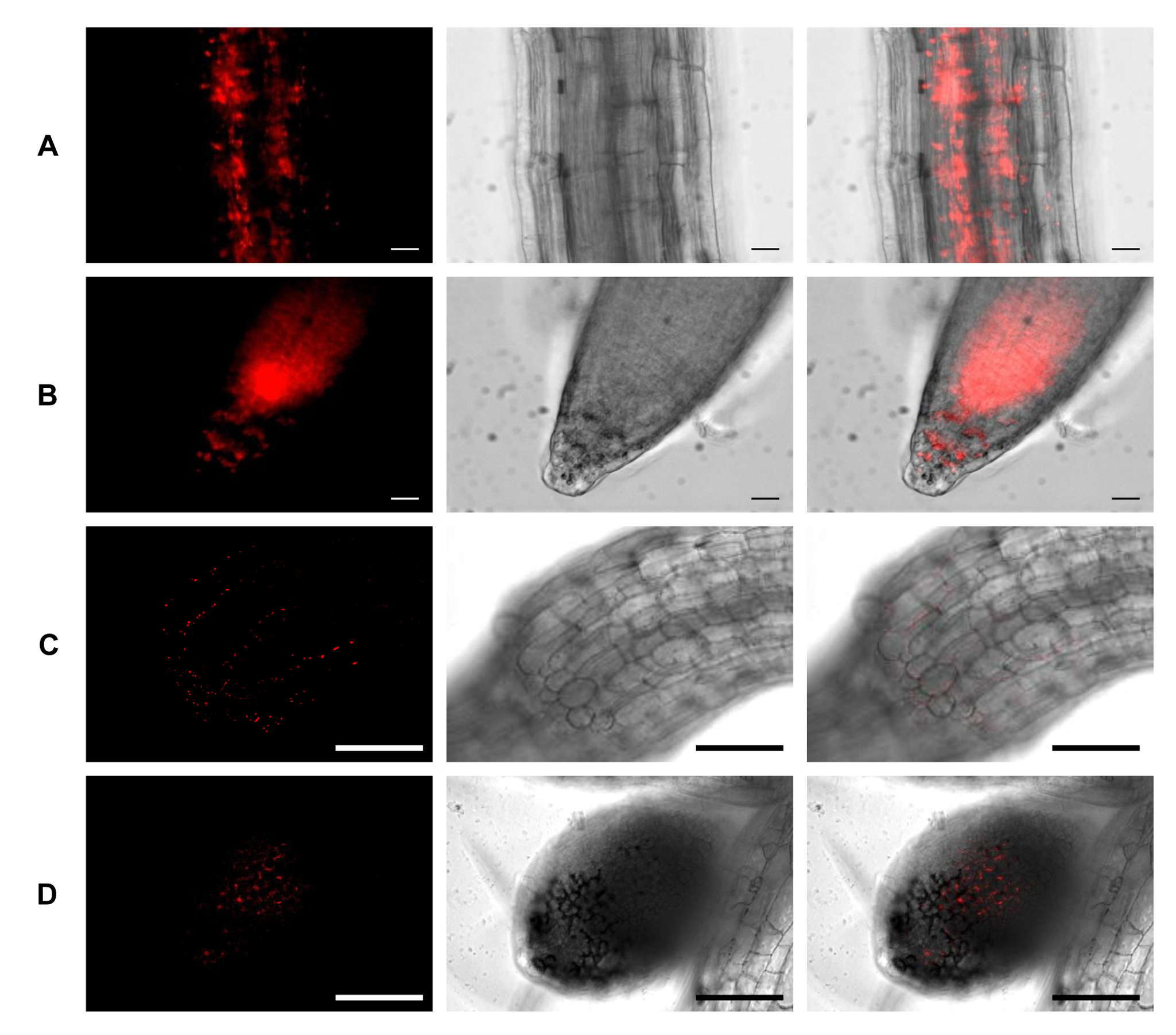

Supplement: Figure S4 — Expression studies of the IPMI SSU1 gene. The complete gene (w/o translation stop codon) including a 432 bp promoter fragment was cloned upstream of the with the RFP gene (details see Material and Methods) and stably integrated into the genome of the ipmi ssu2-1/ipmi ssu3-1 double knockout mutant. Confocal images showed red fluorescence in the following tissues: root (A), root tip (B), basis of hypocotyl (C), and emerging first leaf pair of 5 or 6 day-old seedlings (D). Thick scale bars correspond to 100 µm, thin scale bars to 20 µm. (TIF) [file pone.0091071.s004.tif]

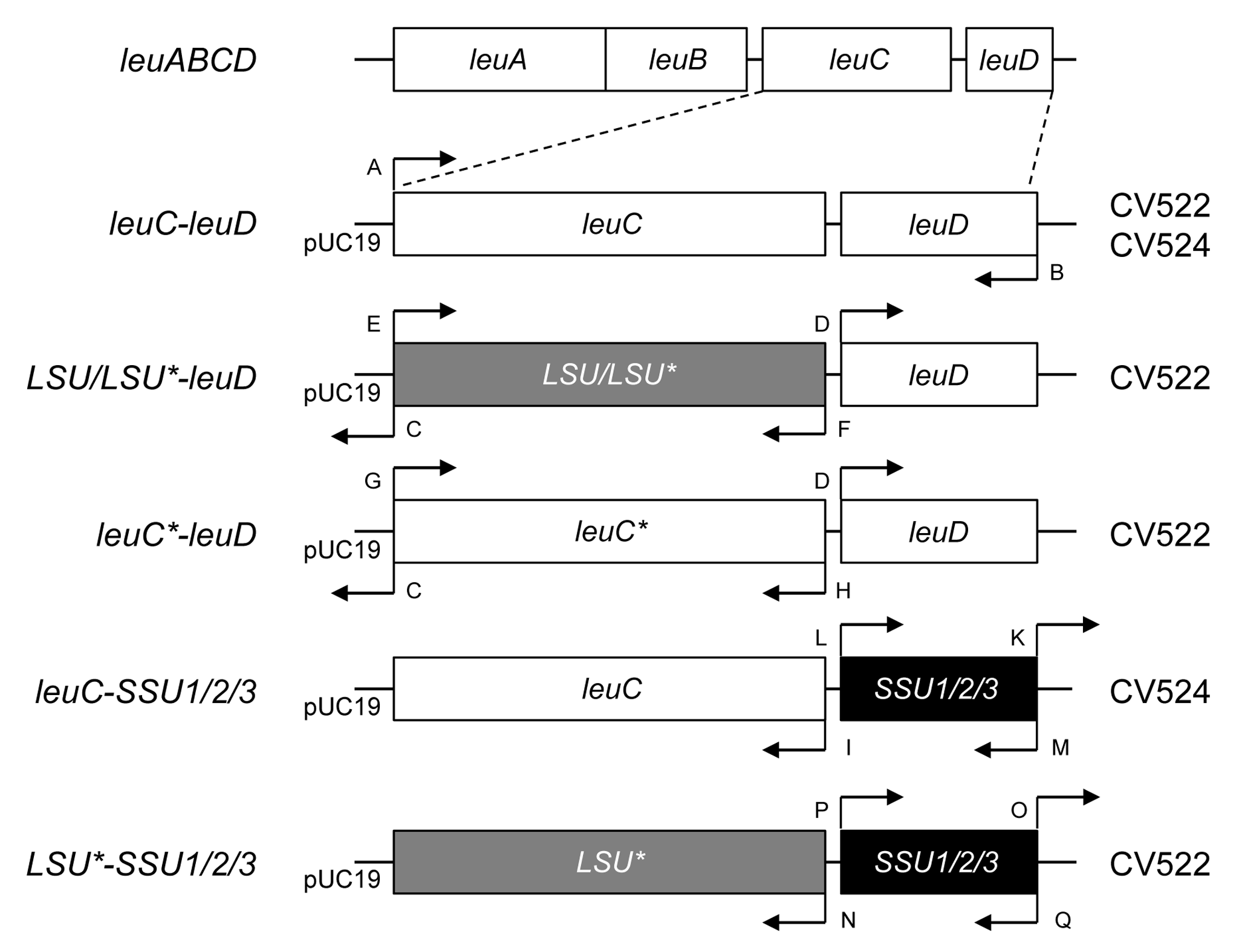

Supplement: Figure S5 — Schematic overview of the constructs used for CV522 (ΔleuC) and CV524 (ΔleuD) for complementation studies. The dicistronic leuC-leuD was amplified from the leu operon (leuABCD) and cloned into pUC19. Afterwards leuC was exchanged by the cDNAs of IPMI LSU1 from Arabidopsis with (LSU) or without 62 amino acids at the N-terminus, corresponding to the predicted plastid targeting sequence (LSU*). E. coli leuC* was cloned in the same way like LSU and LSU*. leuD was replaced by one of the A. thaliana IPMI SSU1, 2 or 3. Positions and orientations of the primers used for PCR are indicated by bent arrows. The following oligonucleotides have been used: A, leuC-comp.H; B, leuD-comp.R; C, LeuCErsatz1 AscI; D, LeuCErsatz2 PacI; E.1, IPMILSU1AscI; E.2, LSU-Chlp.target.AscI; F, IPMILSU2PacI; G, LeuC_AscI; H, LeuC_PacI; I, LeuDErsatz1 AscI; K, LeuDErsatz2 PacI; L.1, IPMISSU1.1AscI; L.2, IPMISSU2.1AscI; L.3, IPMISSU3.1AscI; M.1, IPMISSU1.2PacI; M.2, IPMISSU2.2PacI; M.3, IPMISSU3.2PacI; N, LSU-Xba; O, LeuDErsatz2 SmaI; P.1, IPMISSU1.XbaI; P.2, IPMISSU2.XbaI; P.3, IPMISSU3.XbaI; Q.1, IPMISSU1.SmaI; Q.2, IPMISSU2.SmaI; Q.3, IPMISSU3.SmaI. Oligonucleotide sequences are given in Table S1. (TIF) [file pone.0091071.s005.tif]

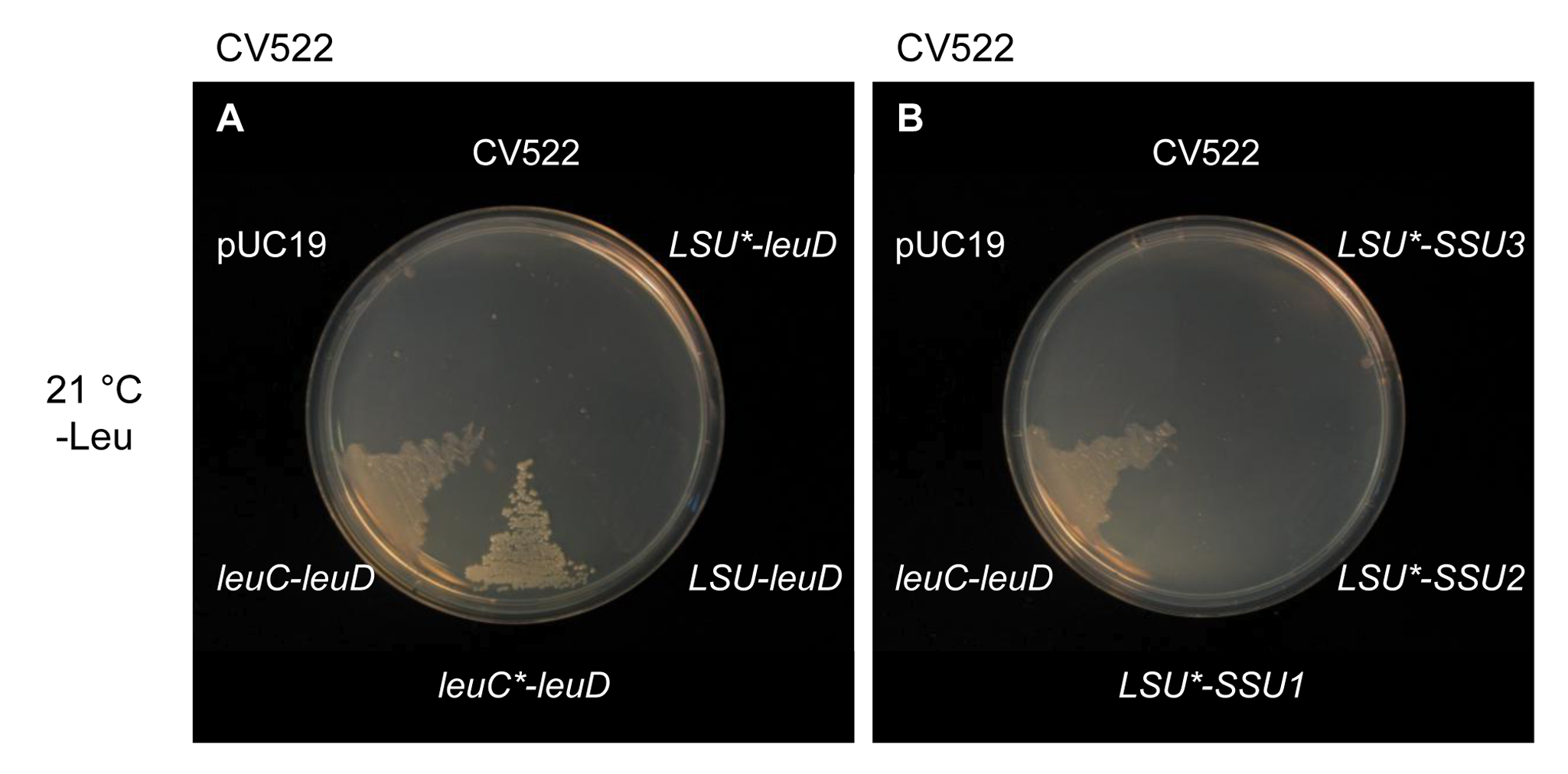

Supplement: Figure S6 — Complementation analysis of CV522 (ΔleuC) at 22°C. Complementation capability of the different IPMI subunits in the Leu-auxotrophic E. coli strain CV522 (ΔleuC, A, B) was tested on minimal medium with 1 mM IPTG at 21°C. The auxotrophic strain was transformed either with empty pUC19, leuC-leuD, or with different combinations of IPMI subunits, leuC*/LSU/LSU*-leuD (A) and LSU*-SSU1/2/3 (B). (TIF) [file pone.0091071.s006.tif]
