# Supplementary material for: The Small Subunit 1 of the Arabidopsis Isopropylmalate Isomerase Is Required for Normal Growth and Development and the Early Stages of Glucosinolate Formation
Source: PLoS One. 2014 Mar 7;9(3):e91071. doi: 10.1371/journal.pone.0091071 (PMC3946710; doi:10.1371/journal.pone.0091071)
Supplement: Table S1 — Oligonucleotide sequences. (PDF) [file pone.0091071.s007.pdf]

Supplemental Table S1

Oligonucleotide sequences

Primer no.	Name	Sequence (5'-3')
		**Establishment of *amiR-SSU1* mutants**
	pRS300A	CTGCAAGGCGATTAAGTTGGGTAAC
	pRS300B	GCGGATAACAATTTCACACAGGAAACAG
	pRS300C	GATATCGAATTCCTGCAGC
	pRS300D	ATGCAGCTGCAGGCTCTAGAACTAGTGGATCC
	B_SSU1_miR-s	GATAACTAAAGCGTAAGAACCAATCTCTCTTTTGTATTCC
	B_SSU1_miR-a	GATTGGTTCTTACGCTTTAGTTATCAAAGAGAATCAATGA
	B_SSU1_miR*s	GATTAGTTCTTACGCATTAGTTTTCACAGGTCGTGATATG
	B_SSU1_miR*a	GAAAACTAATGCGTAAGAACTAATCTACATATATATTCCT
	C_SSU1_miR-s	GATATCACCAGTTGTACACGCATTCTCTCTTTTGTATTCC
	C_SSU1_miR-a	GAATGCGTGTACAACTGGTGATATCAAAGAGAATCAATGA
	C_SSU1_miR*s	GAATACGTGTACAACAGGTGATTTCACAGGTCGTGATATG
	C_SSU1_miR*a	GAAATCACCTGTTGTACACGTATTCTACATATATATTCCT
	D_SSU1_miR-s	GATGATCAAAATACTATCTCCTCTCTCTCTTTTGTATTCC
	D_SSU1_miR-a	GAGAGGAGATAGTATTTTGATCATCAAAGAGAATCAATGA
	D_SSU1_miR*s	GAGAAGAGATAGTATATTGATCTTCACAGGTCGTGATATG
	D_SSU1_miR*a	GAAGATCAATATACTATCTCTTCTCTACATATATATTCCT
		**RT-PCR**
	DTXSC	GACTCGAGTCGACATCGATTTTTTTTTTTTTTTTT
	SSU1-comp.H	ATGCAGCTGCAGAATGGCGGCTTCTCTGCAATCAG
	SSU1-comp.R	ATGCAGGAATTCAGTCAAAATCAAGCAGCAGCAGATGG
	SSU2-comp.H	ATGCAGCTGCAGAACAATGGCGTATTCTCTTCCTAC
	SSU2-comp.R	ATGCAGGAATTCATGGAAGAGGATTAAGCTAATGATGG
	SSU3-comp.H	ATGCAGCTGCAGCAGAACAATGGCGACTTCTCAG
	SSU3-comp.R	ATGCAGGAATTCTCCGATTACATTCAAGCAGAAGG
	At4g13430-Bur.H	GCAGATTATAGATTTGACGTG
	At4g13430-Bur.R	TGTAAGGAGACGCTAAGTAAA
	At2g43090.H	TCCAGAGGAATACGAGAAACTCG
	At2g43090.R	CCTTGTAAGAAGCTGGAAGACCA
	UBC9-H.2	CTACTTCATGTAGCGCAGGAC
	At4g27960-1	TCACAATTTCCAAGGTGCTGC
	At4g27960-2	TCATCTGGGTTTGGATCCGT
	At1g13320-1	TAACGTGGCCAAAATGATGC
	At1g13320-2	GTTCTCCACAACCGCTTGGT
		**Complementation of *E. coli* auxotrophic *leuC* and *leuD* strains**
A	leuC-comp.H	ATGCAGCTGCAGGGGTGTAATCATGGCTAAGACG
B	leuD-comp.R	ATGCAGGAATTCGGTCATTTGACCGGGCAAGG
C	LeuCErsatz1 AscI	ATGCAGGGCGCGCCCTGCAGGCATGC
D	LeuCErsatz2 PacI	ATGCAGTTAATTAACCATGGCAGAGAAATTTATC
E.1	IPMILSU1AscI	ATGCAGGGCGCGCCTCATGGCTTCTGTTATCTCTTCC
E.2	LSU-Chlp.target.AscI	ATGCAGGGCGCGCCTGAAAACTGGGATGACA
F	IPMILSU2PacI	ATGCAGTTAATTAACTACTGCAAGAACTCCCTTG
G	LeuC_AscI	ATGCAGGGCGCGCCTCATGGCTAAGACGTTATACG
H	LeuC_PacI	ATGCAGTTAATTAATTATTTAATGTTGCGAATGTCG

I	LeuDErsatz1 AscI	ATGCAGGGCGCGCCTTATTTAATGTTGCGAATGTC
K	LeuDErsatz2 PacI	ATGCAGTTAATTAACACTGGCCGTCGTTTTAC
L.1	IPMISSU1.1AscI	ATGCAGGGCGCGCCCAATGGCGGCTTCTCTG
M.1	IPMISSU1.2PacI	ATGCAGTTAATTAATCAAGCAGCAGCAGATG
L.2	IPMISSU2.1AscI	ATGCAGGGCGCGCCCAATGGCGTATTCTCTTCC
M.2	IPMISSU2.2PacI	ATGCAGTTAATTAATTAAGCTAATGATGGAATCATTCC
L.3	IPMISSU3.1AscI	ATGCAGGGCGCGCCCAATGGCGACTTCTCAGC
M.3	IPMISSU3.2PacI	ATGCAGTTAATTAATCAAGCAGAAGGAATCATGC
N	LSU-Xba	ATGCAGTCTAGACCCTACTGCAAGAACTCCCTTG
O	LeuDErsatz2 SmaI	ATGCAGGAGCTCCACTGGCCGTCGTTTTAC
P.1	IPMISSU1.XbaI	ATGCAGTCTAGACCCAATGGCGGCTTCTCTG
Q.1	IPMISSU1.SmaI	ATGCAGGAGCTCTCAAGCAGCAGCAGATG
P.2	IPMISSU2.XbaI	ATGCAGTCTAGACCCAATGGCGTATTCTCTTCC
Q.2	IPMISSU2.SmaI	ATGCAGGAGCTCTTAAGCTAATGATGGAATCATTCC
P.3	IPMISSU3.XbaI	ATGCAGTCTAGACCCAATGGCGACTTCTCAGC
Q.3	IPMISSU3.SmaI	ATGCAGGAGCTCTCAAGCAGAAGGAATCATGC
	**Promoter studies with PFP**	
	SSU1-RFP.H	ATGCAGGGATCCCATGGAGGATGTGAAGTTCTGTCTGG
	SSU1-RFP.R	ATGCAGCCCGGGAGCAGCAGCAGATGGAATCATTCC
	**Promoter studies with GUS fusions**	
	SSU1GUS.H	ATGCTAGTCGACAGGATGTGAAGTTCTGTCTGG
	SSU1GUS.R	GAGGGATCCCATTGTTTGTTTCTCTTTGCTG
	SSU2GUS.H	ATGCTAGTCGACGCTGCTTGATTTTGACTTCAGG
	SSU2GUS.R	GAGGGATCCCGCCATTGTTTCTTTACTTTGTTGAG
	SSU3GUS.H	ATGCTAGTCGACCATCTCCTCTGCACGAAAGAAG
	SSU3GUS.R	GAGGGATCCCGCCATTGTTCTGCTACTTC
	**Overlap extension PCR**	
	ssu1comp.H	ATGCAGTCGAGCGGCGCGCCGCTGGACCAGTGATTGATGC
	ssu1-3OE.R	GGGTTTAAAAATTGCTGAGAAGTCGCCATTGTTTGTTTCTCTTTGCTGTGG
	ssu1-3OE.H	CCACAGCAAAGAGAAACAAACAATGGCGACTTCTCAGCAATTTTTAAACCC
	ssu3comp.R	GACTTCGAGTTTAATTAAGATCTCATTTGGTAGATTGC
	ssu3comp.H	ATGCAGTCGAGCGGCGCGCCGCAAGTGGAAGGAAATACG
	ssu3-1OE.R	GCTGATTGCAGAGAAGCCGCCATTGTTCTGCTACTTCTTAAGTGAAGCAGG
	ssu3-1OE.H	CCTGCTTCACTTAAGAAGTAGCAGAACAATGGCGGCTTCTCTGCAATCAGC
	ssu1comp.R	GACTTCGAGTTTAATTAAGCCATTCAGAATTCCAAACG
