# Supplementary material for: The Small Subunit 1 of the Arabidopsis Isopropylmalate Isomerase Is Required for Normal Growth and Development and the Early Stages of Glucosinolate Formation
Source: PLoS One. 2014 Mar 7;9(3):e91071. doi: 10.1371/journal.pone.0091071 (PMC3946710; doi:10.1371/journal.pone.0091071)
Supplement: Table S2 — Amino acid profile in seeds of amiR-SSU1-B plants. (PDF) [file pone.0091071.s008.pdf]

**Supplemental Table S2: Amino acid content in seeds of amiR-SSU1-B lines.**

| Amino Acid | Amino Acid Content [nmol/mg Dry Weight] | |
|---|---|---|
| | Col-0 | amiR-SSU1-B |
| Ala | 1.0 ± 0.3 | 1.0 ± 0.3 |
| Arg | 0.2 ± 0.0 | 0.2 ± 0.1 |
| Asn | 1.6 ± 0.3 | 1.3 ± 0.4 |
| Asp | 1.5 ± 0.2 | 1.1 ± 0.3 * |
| Gln | 0.5 ± 0.2 | 0.4 ± 0.1 |
| Glu | 8.2 ± 1.2 | 7.7 ± 1.4 |
| His | 0.1 ± 0.0 | 0.1 ± 0.0 |
| Ile | 0.2 ± 0.0 | 0.2 ± 0.0 |
| Leu | 0.1 ± 0.0 | 0.1 ± 0.0 |
| Lys | 0.1 ± 0.0 | 0.1 ± 0.0 |
| Met | 0.1 ± 0.0 | 0.1 ± 0.0 |
| Phe | 0.2 ± 0.0 | 0.2 ± 0.0 |
| Pro | 0.2 ± 0.0 | 0.2 ± 0.0 |
| Ser | 0.9 ± 0.2 | 0.8 ± 0.1 |
| Thr | 0.2 ± 0.1 | 0.2 ± 0.0 |
| Trp | 1.2 ± 0.1 | 0.8 ± 0.1 * |
| Tyr | 0.1 ± 0.0 | 0.1 ± 0.0 * |
| Val | 0.3 ± 0.1 | 0.3 ± 0.1 |
| Total | 16.5 ± 1.4 | 14.9 ± 1.8 |

* p-value $p < 0.01$ in a statistical T-Test between Col-0 and amiR-SSU1-B.
