# Supplementary material for: The Small Subunit 1 of the Arabidopsis Isopropylmalate Isomerase Is Required for Normal Growth and Development and the Early Stages of Glucosinolate Formation
Source: PLoS One. 2014 Mar 7;9(3):e91071. doi: 10.1371/journal.pone.0091071 (PMC3946710; doi:10.1371/journal.pone.0091071)
Supplement: Table S3 — Glucosinolate profile in seeds of amiR-SSU1-B plants. (PDF) [file pone.0091071.s009.pdf]

Supplemental Table S3: Glucosinolate content in seeds of amiR-SSU1-B plants.

Glucosinolate	Glucosinolate Content [µmol/g Dry Weight]	
	Col-0	amiR-SSU1-B
4MSOB	1.3 ± 0.5	0.4 ± 0.1 *
5MSOP	0.2 ± 0.1	2.5 ± 0.0 *
6MSOH	0.3 ± 0.1	0.2 ± 0.0 *
7MSOH	1.5 ± 0.3	1.0 ± 0.1 *
8MSOO	9.3 ± 1.2	7.3 ± 0.6 *
4MTB	16.5 ± 3.0	9.2 ± 2.1 *
5MTP	2.2 ± 0.4	1.4 ± 0.1 *
7MTH	8.8 ± 1.2	6.5 ± 0.7 *
8MTO	10.0 ± 1.0	10.0 ± 1.1
3BZO	3.7 ± 0.4	3.1 ± 0.4 *
4BZO	12.9 ± 1.6	11.9 ± 1.2
3OHP	0.4 ± 0.1	0.3 ± 0.1 *
4OHB	2.9 ± 0.3	2.4 ± 0.2 *
I3M	1.3 ± 0.2	1.0 ± 0.2 *
Total	71.4 ± 7.5	57.1 ± 4.1 *

6MSOH , 6-methylsulfinylhexylglucosinolate; 5MTP, 5-methylthiopentylglucosinolate; 7MTH, 7-methylthioheptylglucosinolate; 8MTO 8-methylthiooctylglucosinolate; 3BZO, 3-benzoyloxypropylglucosinolate; 4BZO, 4-benzoyloxybutylglucosinolate; 3OHP, 3-hydroxypropylglucosinolate; 4OHB, hydroxybutylglucosinolate; * p-value $p < 0.01$ in a statistical T-Test between Col-0 and amiR-SSU1-B. Other abbreviations see Table 1.
