# Supplementary material for: The Small Subunit 1 of the Arabidopsis Isopropylmalate Isomerase Is Required for Normal Growth and Development and the Early Stages of Glucosinolate Formation
Source: PLoS One. 2014 Mar 7;9(3):e91071. doi: 10.1371/journal.pone.0091071 (PMC3946710; doi:10.1371/journal.pone.0091071)
Supplement: Table S4 — Relative content of diverse metabolites in seeds of amiR-SSU1-B plants. (PDF) [file pone.0091071.s010.pdf]

**Supplemental Table S4: Relative content of diverse metabolites in seeds of amiR-SSU1-B plants.**

| Metabolite | Relative content** | |
|---|---|---|
| | Col-0 | amiR-SSU1-B |
| quercetin 3-O-rhamnoside | 31491079 ± 7215193 | 51238062 ± 3846241 * |
| quercetin 3-O-rhamnoside 7-O-rhamnoside | 41830653 ± 3566552 | 42728960 ± 4107647 |
| sucrose | 35173891 ± 5357657 | 36993399 ± 8835400 |
| S-methylmethionine | 481119 ± 433273 | 1370996 ± 190909 * |

* p-value $p < 0.01$ in a statistical T-Test between Col-0 and amiR-SSU1-B.

** peak area LC-IONTRAP-MS x 1000
