# Supplementary material for: The Small Subunit 1 of the Arabidopsis Isopropylmalate Isomerase Is Required for Normal Growth and Development and the Early Stages of Glucosinolate Formation
Source: PLoS One. 2014 Mar 7;9(3):e91071. doi: 10.1371/journal.pone.0091071 (PMC3946710; doi:10.1371/journal.pone.0091071)
Supplement: Table S5 — Glucosinolate profile in rosette leaves of an ipmi ssu2-1/ipmi ssu3-1 double knockout mutant. (PDF) [file pone.0091071.s011.pdf]

Supplemental Table S5: Glucosinolate profile in rosette leaves of an *ipmi ssu2-1*/*ipmi ssu3-1* double knockout mutant.

Glucosinolate	Glucosinolate Content [µmol/g Dry Weight]				
	Col-0	*ipmi ssu2-1*	Ws	*ipmi ssu3-1*	*ipmi ssu2-1/ipmi ssu3-1*
3MSOP	1.6 ± 0.2	2.4 ± 0.1	23.8 ± 3.5	25.2 ± 1.4	11.7 ± 0.9 §*
4MSOB	12.4 ± 0.7	16.5 ± 1.0	0.3 ± 0.0	0.3 ± 0.1	7.8 ± 0.5 §*
5MSOP	n.d.	0.6 ± 0.0	n.d.	n.d.	n.d. §
7MSOH	0.5 ± 0.0	0.6 ± 0.1	0.1 ± 0.0	0.1 ± 0.0	n.d. §*
8MSOO	2.1 ± 0.2	1.5 ± 0.2	2.4 ± 0.5	2.6 ± 0.1	n.d. §*
3MTP	0.1 ± 0.0	n.d.	0.6 ± 0.1	0.4 ± 0.0	n.d.*
4MTB	0.9 ± 0.0	0.8 ± 0.3	n.d.	n.d.	0.4 ± 0.1 §*
I3M	2.1 ± 0.2	2.6 ± 0.2	2.1 ± 0.4	2.8 ± 0.2	3.1 ± 0.4 §
1MOI3M	0.7 ± 0.2	0.7 ± 0.2	0.4 ± 0.2	0.2 ± 0.1	0.6 ± 0.2 *
4MOI3M	0.5 ± 0.1	0.6 ± 0.1	0.2 ± 0.0	0.3 ± 0.0	0.4 ± 0.1 §*
Total	20.9 ± 1.4	26.4 ± 1.5	30.1 ± 4.4	31.9 ± 1.4	24.0 ± 1.5 §*

§ p-value $p < 0.01$ in a statistical T-Test between *ipmi ssu2-1* and *ipmi ssu2-1/ipmi ssu3-1*.

* p-value $p < 0.01$ in a statistical T-Test between *ipmi ssu3-1* and *ipmi ssu2-1/ipmi ssu3-1*.

Abbreviations see legends of Table 1 and Supplemental Table S3.
