# Supplementary material for: The Small Subunit 1 of the Arabidopsis Isopropylmalate Isomerase Is Required for Normal Growth and Development and the Early Stages of Glucosinolate Formation
Source: PLoS One. 2014 Mar 7;9(3):e91071. doi: 10.1371/journal.pone.0091071 (PMC3946710; doi:10.1371/journal.pone.0091071)
Supplement: Table S6 — Glucosinolate content in seeds of pSSU1:SSU3 and pSSU3:SSU1 lines. (PDF) [file pone.0091071.s012.pdf]

Supplemental Table S6: Glucosinolate content in seeds of pSSU1:SSU3 and pSSU3:SSU1 lines.

Glucosinolate	Glucosinolate Content [µmol/g Dry Weight]				
	Col-0	Ws	*ipmi ssu2-1/ipmi ssu3-1*	*ipmi ssu2-1/ipmi ssu3-1/* pSSU3:SSU1	*ipmi ssu2-1/ipmi ssu3-1/* pSSU1:SSU3
3MSOP	n.d.	3.1 ± 1.3	n.d.	3.8 ± 1.6 §	n.d.
4MSOB	0.6 ± 0.1	0.1 ± 0.0	0.5 ± 0.1	0.9 ± 0.1 §	1.3 ± 0.3 *
5MSOP	0.1 ± 0.0	n.d.	n.d.	n.d.	n.d.
6MSOH	0.1 ± 0.0	n.d.	n.d.	n.d.	n.d.
7MSOH	1.0 ± 0.2	0.5 ± 0.1	n.d.	n.d.	n.d.
8MSOO	7.5 ± 1.0	7.0 ± 1.2	n.d.	n.d.	n.d.
3MTP	n.d.	25.8 ± 3.4	0.8 ± 0.2	8.0 ± 2.5 §	0.3 ± 0.1 *
4MTB	15.6 ± 2.1	1.58 ± 0.1	4.6 ± 0.5	11.5 ± 1.9 §	26.1 ± 3.0 *
5MTP	1.9 ± 0.3	n.d.	n.d.	0.6 ± 0.1 §	1.3 ± 0.1 *
7MTH	6.8 ± 1.0	3.52 ± 0.3	n.d.	n.d.	n.d.
8MTO	9.2 ± 1.0	14.53 ± 0.7	n.d.	n.d.	n.d.
3BZO	4.1 ± 0.2	9.85 ± 0.8	22.0 ± 1.0	24.5 ± 1.7 §	12.5 ± 1.3 *
4BZO	14.6 ± 1.6	0.17 ± 0.0	5.7 ± 0.2	3.1 ± 0.6 §	11.3 ± 0.6 *
3OHP	0.6 ± 0.2	3.12 ± 0.4	9.2 ± 0.8	16.5 ± 1.3 §	5.6 ± 0.7 *
4OHB	3.7 ± 0.6	n.d.	2.8 ± 0.4	2.9 ± 0.5	12.4 ± 0.9 *
I3M	1.1 ± 0.3	0.41 ± 0.1	0.7 ± 0.1	0.8 ± 0.1	0.5 ± 0.0 *
Total	66.9 ± 4.3	69.70 ± 4.7	46.0 ± 2.2	72.6 ± 6.4 §	71.3 ± 5.2 *

§ p-value $p < 0.01$ in a statistical T-Test between *ipmi ssu2-1/ipmi ssu3-1* and *ipmi ssu2-1/ipmi ssu3-1*/pSSU3:SSU1.
* p-value $p < 0.01$ in a statistical T-Test between *ipmi ssu2-1/ipmi ssu3-1* and *ipmi ssu2-1/ipmi ssu3-1*/pSSU1:SSU3.
Abbreviations see legends of Table 1 and Supplemental Table S3.
