# Supplementary material for: The Small Subunit 1 of the Arabidopsis Isopropylmalate Isomerase Is Required for Normal Growth and Development and the Early Stages of Glucosinolate Formation
Source: PLoS One. 2014 Mar 7;9(3):e91071. doi: 10.1371/journal.pone.0091071 (PMC3946710; doi:10.1371/journal.pone.0091071)
Supplement: Protocol S1 — Metabolite analysis by LC-MS/MS. (DOCX) [file pone.0091071.s013.docx]

**Supplemental Protocol S1**

**Metabolite analysis by LC-MS/MS**

To measure amino acids by LC-MS/MS leaves were freeze-dried and ground to a fine powder. Amino acids were extracted from 10 mg or 20 mg freeze-dried and pulverized leaf material with 1 ml of 80 % methanol (v/v) and the resulting extract was diluted in a ratio of 1:10 (v/v) in water containing the ^13^C, ^15^N labeled algal amino acid mix (Isotec, Miamisburg, US, 10µg/mL). Amino acids in the diluted extracts were directly analyzed by LC-MS/MS as outlined before [[15](#_ENREF_11)] with the modifications that a different elution profile (0-1 min, 3 % B in A; 1-2.7 min, 3-100 % B in A; 2.7-3 min 100 % B and 3.1-6 min 3 % B in A) was applied. Mobile phase flow rate was 1.1 ml/min and an API5000 mass spectrometer (Applied Biosystems) was used in multiple reaction monitoring (MRM) mode. Levels of glucosinolates were measured according to a previously established protocol [[16](#_ENREF_12)]. To identify further metabolites differing between amiR-SSU1-B plants and wild type, the flow-through fractions recovered from the anion exchange solid-phase extraction performed in the course of glucosinolate analysis of leaves were compared by LC-MS using a Bruker Esquire 6000 ion trap mass spectrometer (Bruker Daltonics, Bremen, Germany) as previously described [[5](#_ENREF_2)] except that mobile phases were 0.2 % formic acid (v:v) (A) and acetonitrile (B), starting with 100 % A for 5 min, followed by a gradient to 45 % B in 15 min. The subtraction of the mass spectrometer total ion chromatogram of wild-type plants from that of the amiR-SSU1-B plant was done using the software package Metabolite Detect 1.1, BrukerDaltonics, Bremen, Germany). For relative quantification peak areas of the respective extracted ion traces for different compounds were extracted as follows: positive ionization mode: S-methylmethionine m/z 102+164; negative ionization mode: 2-(2'-methylsulfinyl)ethylmalate m/z 223; 2-(3'-methylsulfinyl)propylmalate m/z 237; isopropylmalate m/z 175; saccharose m/z 341+377+387; m/z 591; kaempferol 3-O-glucoside 7-O-rhamnoside m/z 593; quercetin 3-O-rhamnoside m/z 447; quercetin 3-O-rhamnoside 7-O-rhamnoside m/z 593.
