# Supplementary material for: The Small Subunit 1 of the Arabidopsis Isopropylmalate Isomerase Is Required for Normal Growth and Development and the Early Stages of Glucosinolate Formation
Source: PLoS One. 2014 Mar 7;9(3):e91071. doi: 10.1371/journal.pone.0091071 (PMC3946710; doi:10.1371/journal.pone.0091071)
Supplement: Protocol S2 — Analysis of isopropylmalate and intermediates of glucosinolate biosynthesis. (DOCX) [file pone.0091071.s014.docx]

**Supplemental Protocol S2**

**Analysis of isopropylmalate and intermediates of glucosinolate biosynthesis**

To determine the relative contents of isopropylmalate, 2-(2'-methylsulfinyl)ethylmalate, and 2-(3'-methylsulfinyl)propylmalate an LC-MS/MS analysis was performed. Leaf extracts obtained during glucosinolate analysis were separated by chromatography on a Zorbax Eclipse XDB-C18 column (50 x 4.6 mm, 1.8 µm, Agilent Technologies, Germany) using an Agilent 1200 HPLC system (Agilent Technologies, Böblingen, Germany). Formic acid (0.05 %) in water and acetonitrile were employed as mobile phases A and B respectively. The elution profile was: 0 - 0.5 min, 5 % B in A; 0.5 – 3 min, 5 - 58 % B in A; 3.1 – 4 min 100 % B and 4.1 - 6.5 min 5 % B in A. The mobile phase flow rate was 1.1 ml/min. The column temperature was maintained at 25 °C. The liquid chromatography was coupled to an API 3200 tandem mass spectrometer (Applied Biosystems, Darmstadt, Germany) equipped with a Turbospray ion source operated in negative ionization mode. The ionspray voltage was maintained at -4500 eV. The turbo gas temperature was set at 700 °C. Nebulizing gas was set at 60 psi, curtain gas at 25 psi, heating gas at 60 psi and collision gas at 7 psi. Multiple reaction monitoring (MRM) was used to monitor analyte parent ion → product ion: m/z 175 →115 (collision energy (CE)-20 V; de-clustering potential (DP) -30 V) for isopropylmalate; m/z 223 →163 (CE -20 V; DP -30 V) for 2-(2'-methylsulfinyl)ethylmalate; m/z 237 →177 (CE -20 V; DP -30 V) for 2-(3'-methylsulfinyl)propylmalate. Both Q1 and Q3 quadrupoles were maintained at unit resolution. Analyst 1.5 software (Applied Biosystems, Darmstadt, Germany) was used for data acquisition and processing. Relative quantification of the compounds was based on peak areas of the MRM chromatograms.
