# Supplementary material for: The Small Subunit 1 of the Arabidopsis Isopropylmalate Isomerase Is Required for Normal Growth and Development and the Early Stages of Glucosinolate Formation
Source: PLoS One. 2014 Mar 7;9(3):e91071. doi: 10.1371/journal.pone.0091071 (PMC3946710; doi:10.1371/journal.pone.0091071)
Supplement: Protocol S3 — Complementation studies leucine auxotrophic E. coli strains. (DOCX) [file pone.0091071.s015.docx]

**Supplemental Protocol S3**

**Complementation studies leucine auxotrophic *E. coli* strains**

Leucine auxotrophic *E. coli* strains lacking intact *leuC* (CV522) and *leuD* (CV524) were obtained from the *E.coli* Genetic Stock Center (New Haven). For complementation, the *leuC* and *leuD* genes were amplified from *E.coli* DH5α with primers leuC-comp.H/leuD-comp.R and cloned into the *Pst*I/*Eco*RI restriction sites in pUC19 (pleuC-leuD). To replace *leuD* by the different small IPMI subunits from *Arabidopsis* the pleuC-leuD backbone was amplified with the primers LeuDErsatz1PacI/LeuDErsatz2AscI, which removed *leuD* and introduced *Asc*I and *Pac*I restriction sites into the PCR product. The cDNAs of the various small IPMI subunits from *Arabidopsis* were amplified with the oligonucleotide pairs IPMISSU1.1AscI/IPMISSU1.2PacI (*IPMI SSU1*), IPMISSU2.1AscI/IPMISSU2.2PacI (*IPMI SSU2*) and IPMISSU3.1AscI/IPMISSU3.2PacI (*IPMI SSU3*) and cloned downstream of the *leuC* into pleuC-leuD using the restriction sites *Asc*I and *Pac*I generating plasmids pleuC-SSU1, pleuC-SSU2 and pleuC-SSU3. The same experimental strategy was used to replace *leuC* by IPMI LSU1 from *Arabidopsis*. The pleuC-leuD plasmid was amplified with the primers LeuCErsatz1PacI/LeuCErsatz2AscI, which removed *leuC* and introduced *Asc*I and *Pac*I restriction sites into the PCR product. The cDNA of *IPMI LSU1* from *Arabidopsis* was amplified with the primer pairs IPMILSU1AscI/IPMILSU12PacI or LSU-Chlp.target.AscI/IPMILSU1PacI. The latter generated a IPMI LSU1 cDNA product without potential chloroplast targeting sequence (corresponding to amino acid position 1 to 186), which we designated LSU*. Both PCR products were cloned upstream of *leuD* using the restriction sites *Asc*I and *Pac*I to generate plasmids pLSU-leuD and pLSU*-leuD, respectively. Likewise, *leuC* was amplified with the primers LeuC_AscI/LeuC_PacI and analogously cloned upstream of *leuD* (pleuC*-leuD). To combine the different small IPMI subunits from *Arabidopsis with IPMI* LSU* the pLSU*-leuD vector backbone was amplified with the primers LSU-Xba/LeuDErsatz2SmaI, which introduced *Xba*I and *Sma*I restriction sites. This linear vector was ligated to cDNAs from the different IPMI SSU with oligonucleotide pairs IPMISSU1.XbaI/IPMISSU1.2SmaI (*IPMI SSU1*), IPMISSU2.1XbaI/IPMISSU2.2SmaI (*IPMI SSU2*) and IPMISSU3.1XbaI/IPMISSU3.2SmaI (*IPMI SSU3*).

For the complementation tests, the different constructs were transformed into CV522 and/or CV524 using standard procedures. After cultivation on solid LB media over night, colonies containing the respective constructs were transferred onto M9 minimal media or onto M9 with 2 mM Leu as control. Bacteria were incubated at 37 °C or 21 °C for 16 to 18 h or 48h after transformation with pleuC*-leuD.
